# Platinum(II)‐Based Optical Probes for Imaging Quadruplex DNA Structures via Phosphorescence Lifetime Imaging Microscopy

**DOI:** 10.1002/anie.202310402

**Published:** 2023-09-08

**Authors:** Jessica Berrones Reyes, Peter S. Sherin, Amrita Sarkar, Marina K. Kuimova, Ramon Vilar

**Affiliations:** ^1^ Department of Chemistry Imperial College London White City Campus 82 Wood Lane London W12 0BZ UK

**Keywords:** Cellular Imaging, PLIM, Phosphorescence, Platinum, Quadruplex DNA

## Abstract

G‐quadruplex DNA is a non‐canonical structure that forms in guanine‐rich regions of the genome. There is increasing evidence showing that G‐quadruplexes have important biological functions, and therefore molecular tools to visualise these structures are important. Herein we report on a series of new cyclometallated platinum(II) complexes which, upon binding to G‐quadruplex DNA, display an increase in their phosphorescence, acting as switch‐on probes. More importantly, upon binding to G‐quadruplexes they display a selective and distinct lengthening of their emission lifetime. We show that this effect can be used to selectively visualise these structures in cells using Phosphorescence Lifetime Imaging Microscopy (PLIM).

## Graphical abstract

New platinum(II)‐based optical probes for G‐quadruplex DNA are reported, which switch‐on their emission intensity and, more importantly, they display a selective and distinct lengthening of their emission lifetime when bound to G‐quadruplex DNA. We show that this effect can be used to selectively visualise these structures in cells using Phosphorescence Lifetime Imaging Microscopy.

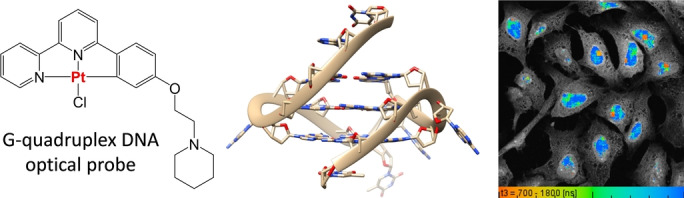

## Introduction

Over the past few years, it has become increasingly clear that non‐duplex DNA secondary structures play key regulatory functions in cells. When DNA is processed during replication and transcription, the canonical double helix unfolds with the resulting single‐stranded regions being able to self‐assemble into non‐duplex structures. One of these—the guanine‐quadruplex (G4) DNA structure—has received particular attention due to its high stability and increasing evidence of its importance in biological processes.^[1]^ G4 structures form in guanine‐rich regions of DNA via Hoogsteen base pairing between guanines. Interestingly, early bioinformatic studies showed that guanine‐rich sequences in the human genome (as well as that of several other organisms) are not randomly distributed but concentrated in regulatory regions as well as at telomeres.^[2]^ Subsequent in vitro studies showed that there are over 700,000 sequences in the human genome with the potential to fold into G4 structures.^[3]^ Further studies in human cells using chromatin immunoprecipitation and high‐throughput sequencing, narrowed this down to *ca*. 10,000 sequences that fold into G4s, with most of these sequences found in regulatory, nucleosome‐depleted regions.^[4]^

Considering the above, there have been significant efforts to develop probes that allow for the visualization of G4s in cells. One of these approaches relies on immunostaining of fixed cells using antibodies with high affinity towards G4 structures.^[5]^ An alternative approach has been to use small‐molecule optical probes that can selectively image G4s over other DNA structures.^[6]^ However, the design of such optical probes is challenging, since they require to have high levels of selectivity for G4s over other DNA structures, due to the transient nature and the low level of G4s in cells at any given point in time, as compared to duplex DNA. While hundreds of probes with such fluorescence switch‐on properties have been developed for in vitro studies,[^6a^, ^7^] only three approaches have been shown to successfully operate in cellular environments. These are: i) probes that shift their emission wavelength significantly upon G4 binding, but not upon binding to other topologies;^[8]^ ii) probes suitable for single molecule microscopy;^[9]^ iii) probes that selectively change their emission lifetime upon interaction with G4 DNA.^[10]^ All the above strategies are successful in detecting small quantities of G4s, over the background of the excess of a duplex DNA, due to a ratiometric nature of these signals, that allows to detect G4s in a concentration‐independent manner. Over the past few years, we focused on the lifetime‐based detection approach, whereby a fluorescent probe reduces its conformational flexibility upon binding to G4s, therefore enhancing its fluorescence lifetime, detectable by Fluorescence Lifetime Imaging Microscopy (FLIM), in live cells. Thus, FLIM provided us[^10a^, ^10b^] and others[^10d^, ^10e^, ^11^] an elegant way of detecting an increase or decrease in G4 presence under varying biological stimuli in live cells. While this has proven to be an excellent method to visualise G4s, we were interested in exploring probes with significantly longer emission lifetimes to avoid potential interference from autofluorescence from naturally occurring fluorophores. Thus, herein we present a new family of high‐affinity phosphorescent probes for G4 detection based on platinum(II) complexes bearing cyclometallated phenyl‐bipyridines. We demonstrate that upon DNA binding, these probes switch‐on their phosphorescence intensity and, uniquely, display DNA‐topology dependent phosphorescence lifetimes, in the microsecond range, setting them apart from the interference from most fluorophores. This unique observation allowed us to perform Phosphorescence Lifetime Imaging Microscopy (PLIM) studies to directly detect G4 foci in cancer cells. To the best of our knowledge this is the first time that G4 DNA structures are selectively imaged in cells using PLIM.

## Results and Discussion

### Synthesis and characterization of platinum(II)‐complexes

**Scheme 1:**
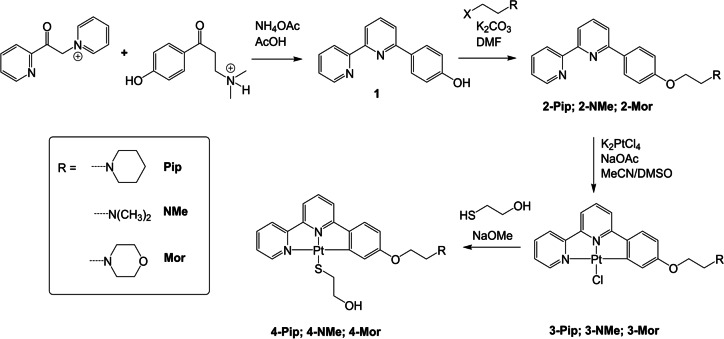
Synthesis of bipyridine‐based ligands and the corresponding cyclometallated platinum (II) complexes.

Square planar, cyclometallated platinum(II) complexes containing polyaromatic ligands have attracted significant interest due to their unique photophysical properties (see for example the extensive studies by Che,^[12]^ Williams^[13]^ and Yam,^[14]^ amongst others^[15]^). Specifically, they display phosphorescence intensity and lifetime, which are highly dependent on the immediate environment of the probe. Based on the previous work showing that platinum(II) terpyridines containing amine substituents display good affinity and selectivity for G4 DNA,^[16]^ we designed the cyclometallating ligands shown in Scheme 1. The resulting square‐planar platinum(II) complexes with these ligands retain the geometrical features of the terpyridine analogues (i.e. delocalised π‐system of the right dimensions to stack on top of the G4 tetrad, and their amine substituents which increase aqueous solubility and DNA interactions). In addition, as indicated above, the N^N^C coordination, should lead to phosphorescent platinum(II) cyclometallated complexes.

The core aromatic system 6‐(4′′‐hydroxy‐phenyl)‐2,2′‐bipyridine (**1**) was prepared following a previously described synthetic procedure^[17]^ and reacted further to synthesise **2‐Pip**, **2‐NMe** and **2‐Mor** (where Pip=piperidine; NMe=dimethyl amine; Mor=morpholine—see Scheme 1). The ligands were then reacted with K_2_PtCl_4_ and complexes **3‐Pip**, **3‐NMe** and **3‐Mor** were obtained in high yields (91–98 %). These new platinum(II) cyclometallated complexes were fully characterized by NMR spectroscopy, mass spectrometry and their identity confirmed by elemental analysis (see Figures S3–S13 and S29–S31). The ^1^H NMR spectra of the three complexes showed the expected ten aromatic signals, with the one closest to the platinum centre displaying satellites due to ^195^Pt coupling. The formation of a Pt−C bond via cyclometallation was confirmed by the change in the coupling pattern of the corresponding phenyl ring, as well as the loss of one proton in the total integration as compared to the free ligand. Mass spectra of the three complexes (with m/z of 590, 550 and 592 [M+H^+^] for **3‐Pip**, **3‐NMe** and **3‐Mor** respectively) and elemental analyses confirmed the proposed structures.

In order to improve the solubility of the platinum(II) complexes for biophysical and biological studies, we substituted the chloride ligand with mercaptoethanol, as was previously demonstrated for platinum(II) terpyridine complexes.[^16d^, ^18^] Thus, compounds **4‐Pip**, **4‐NMe** and **4‐Mor** (see Scheme 1) were prepared by reacting the corresponding platinum(II)‐chloride complex (i.e. **3‐Pip**, **3‐NMe** and **3‐Mor** respectively) with mercaptoethanol in the presence of sodium methoxide (see Figures S14–S24 and S32–S34 for spectroscopic and analytical data). The ^1^H NMR spectra displayed the expected ten aromatic peaks and two additional signals (H‐α and H‐β) in the aliphatic region at 3.72 and 3.00 ppm associated to mercaptoethanol (with the resonance associated to H‐α displaying satellites due to ^195^Pt coupling). The mass spectra of these three new complexes (with m/z of 631, 591 and 633 [M+H^+^] for **4‐Pip**, **4‐NMe** and **4‐Mor** respectively) and elemental analyses confirmed the proposed structures.

**Figure 1:**
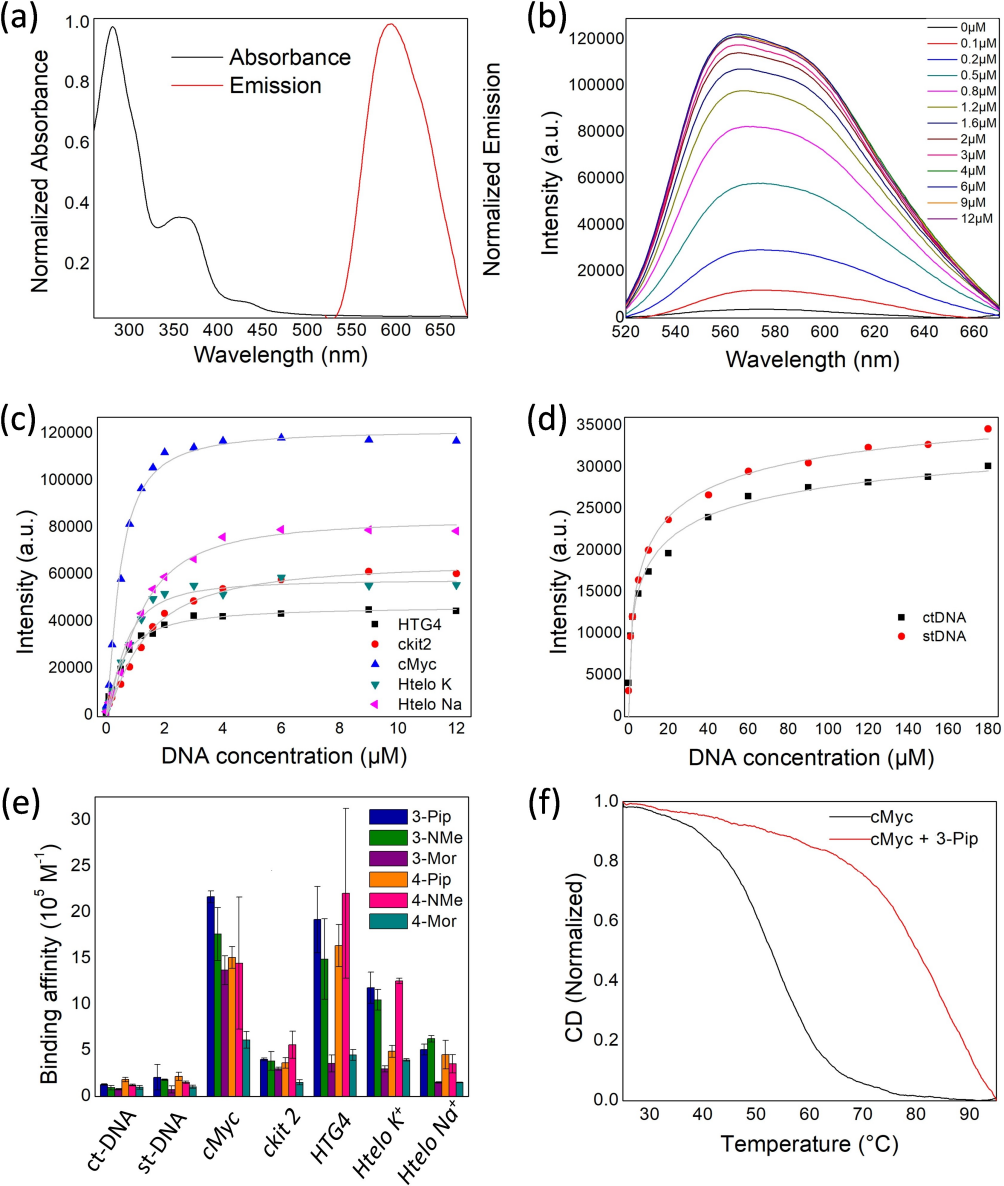
a) Normalized absorbance and emission (λ_ex_=350 nm) of **3‐Pip**, recorded in CH_2_Cl_2_; b) Emission spectra of **3‐Pip** upon addition of increasing amounts of *c‐Myc* DNA (for full titration data, see Supporting Information; c) Titration curves of **3‐Pip** (1 μM) upon addition of different quadruplex DNA structures (*c‐Myc*, *ckit‐2*, *HTG4*, *HTelo*(K^+^) and *HTelo*(Na^+^), and d) duplex DNA (ct‐DNA and st‐DNA) (λ_em_=580 nm); e) summary of the affinity constants (*K*_a_) of platinum(II) complexes for different topologies of DNA determined by emission titrations; f) Circular Dichroism (CD) melting curves of *c‐Myc* and *c‐Myc* upon addition of **3‐Pip**. The DNA solutions (both G4 and duplex) used for the titrations described in this Figure were prepared in lithium cacodylate buffer at pH 7.3 and supplemented with either K^+^ or Na^+^ as described in the experimental details (see Supporting Information).

The UV/Vis absorption spectra for the platinum(II) complexes of the type **3‐R** and **4‐R** (all of which are new) were recorded in aqueous solution and in a variety of organic solvents (see Figures S35–40). The six complexes exhibit an intense absorption band centered at *ca*. 280 nm with a further broad absorption extending from 330 to 500 nm. This is consistent with the UV/Vis spectra previously reported for platinum(II) complexes coordinated to C^N^N cyclometallating ligands, where the high intensity region below 370 nm is normally assigned to the intra‐ligand (^1^IL) π→π* transitions and the weaker absorption band around 430 nm can be assigned to ^1^MLCT (5d)Pt→π*(L) transitions (where MLCT refers to metal‐to‐ligand charge transfer).^[12c]^ Their emission spectra were also recorded (Figure 1 and S25–S40) displaying the highest emission intensity in CHCl_3_ and CH_2_Cl_2_, with the peak centered at *ca*. 580 nm (λ_ex_=350 nm). Whereas the emission intensity in all other solvents (i.e. CH_3_CN, CH_3_OH, DMSO and aqueous media) was very weak (Table S2). Interestingly, the addition of DNA to aqueous solutions of the platinum(II) complexes led to a significant increase in the intensity of the emission, which is consistent with previous reports for other platinum(II) cyclometallated compounds.^[12]^ Thus, the new complexes herein reported are promising switch‐on probes for DNA.

### DNA binding studies

Increasing amounts of five different G4 DNA structures (*c‐Myc*, *ckit‐2*, *HTG4*, *HTelo*(K^+^) and *HTelo*(Na^+^)—see Supporting Information for sequences) and two duplexes, Calf Thymus (ct‐DNA) and Salmon Testes (st‐DNA) were added, while maintaining a constant concentration of the probes. These sequences are commonly used to test G4 binding ligands not only due to their biological relevance but also their different topologies with *c‐Myc* and *ckit‐2* being parallel, *HTG4* and *HTelo*(K^+^) mixed/hybrid and *HTelo*(Na^+^) antiparallel. Upon each successive addition of DNA, a gradual increase of the emission intensity was observed without any significant shift in the emission wavelength (see Figures 1b and S46). The switch‐on behavior of the probes was significantly more pronounced when adding G4 DNA as compared to duplex DNA, especially for compounds **3‐R** (see Figure 1c, 1d and S46). From these titrations, the DNA binding affinities of the complexes for the different DNA topologies were determined (see Figure 1e and Table S3). Overall, these data shows that all compounds have a preference in binding for G4 DNA structures over duplex DNA. We also verified that no significant unwanted substitution of the chloride ligand occurs in the presence of guanosine at the conditions and timescales of our experiments, Figures S42–44.

Within the G4 structures, the compounds generally have higher affinity for *c‐Myc* followed by the two telomeric sequences with different flanking bases, *HTG4* and *HTelo*(K^+^). Interestingly the affinities across the board for *c‐kit2* and *HTelo*(Na^+^) are between 3 and 10 times lower than for the other G4 structures. However, it is not possible to conclude whether this is due to overall G4 topology since *c‐kit2* and *HTelo*(Na^+^) are parallel and antiparallel, respectively—although as compared to *c‐Myc* (the other parallel sequence under study) the structure of *ckit2* (see PDB 2KYP)^[19]^ shows that an adenine and a cytosine base π–π stack strongly with one of the external G‐tetrads which may reduce the interaction between the platinum(II) complexes and the G4.

The binding affinity was generally higher for the complexes containing either piperidine or dimethyl amine as substituents, as compared to those complexes with morpholine. This can be rationalized by the differences in pKa between the three amines, with morpholine being the less likely to be protonated under physiological conditions (pKa values for methyl‐morpholine, methyl‐piperidine and ethyl‐di‐methyl amine are 7.4, 10.1 and 10.2 respectively).^[20]^ Generally, the coordination of mercaptoethanol to the platinum(II) center appears to reduce the affinity of the complexes with respect to the corresponding Cl‐substituted compounds, although there are some examples where the opposite is observed, e.g. **3‐NMe** has a lower affinity for *HTG4* than **4‐NMe**.

To further probe the interaction of the new platinum(II) complexes with G4 DNA, we performed circular dichroism (CD) melting studies, which provide a measure of the thermal stabilization of G4 DNA induced by the addition of a binder. For this, we chose two of the compounds (**3‐Pip** and **3‐NMe**, which displayed the highest G4 affinities overall), and two sequences: *HTelo*(Na^+^) and *c‐Myc* G4 DNA. These two sequences were selected as representative examples of a parallel (*c‐Myc*) and antiparallel (*HTelo(Na^+^)*) G4 structures. The CD melting studies (Figure 1f and S48) showed that **3‐Pip** and **3‐NMe** provide high stabilisation upon binding to *c‐Myc* G4 DNA (with ΔT_m_=28.3 and 30 °C respectively), and only weak stabilization when bound to *HTelo* (Na^+^) (with ΔT_m_=2.8 and 3.6 °C respectively). These results are consistent with the affinity constants determined via emission titrations.

**Figure 2:**
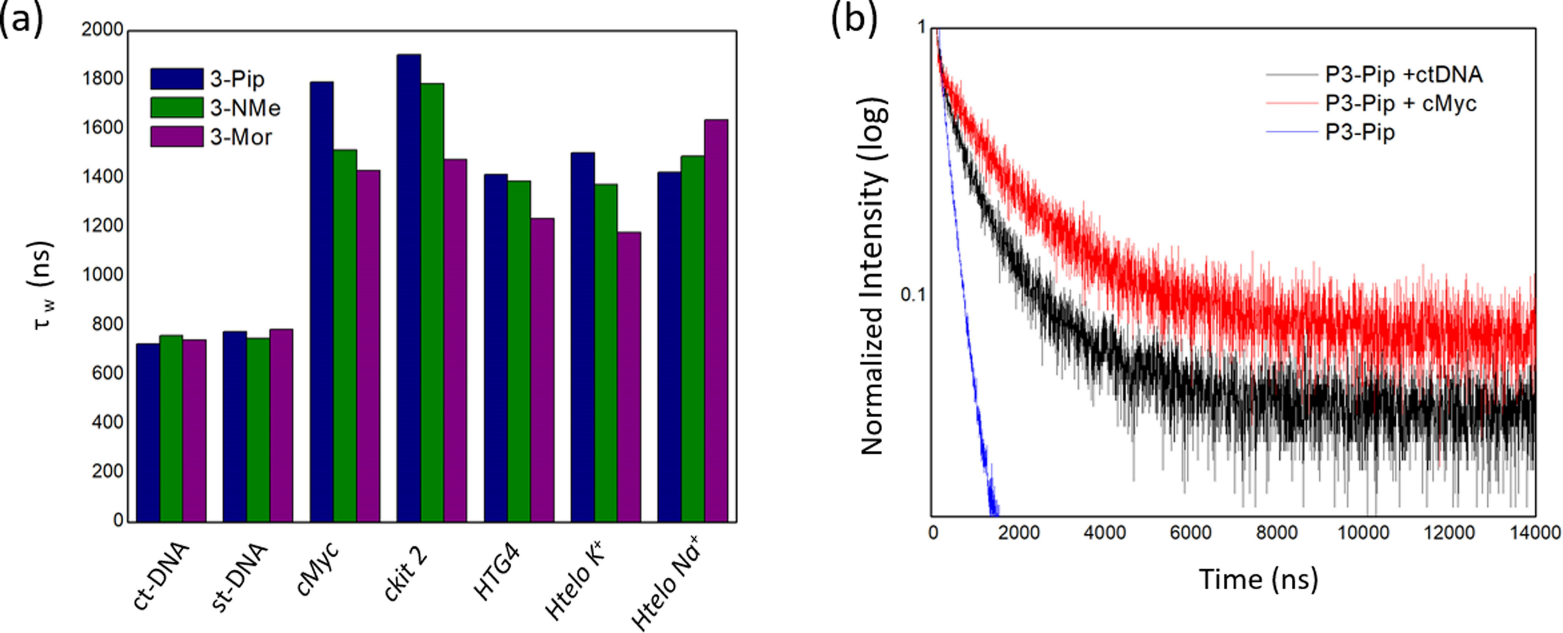
a) Intensity‐weighted average lifetime (τ_w_) of the bi‐exponential decays of **3‐Pip**, **3‐NMe** and **3‐Mor** following the additions of the excess of different DNA sequences; b) Time resolved fluorescence decays of **3‐Pip**, upon addition of 15×equivalents of G4 (*c‐Myc*), and of 15 bp equivalents of dsDNA (ct‐DNA). The data shown in a) was recorded using 1 μM of probe plus 10 equivalents (G4 DNA) or 100 equivalents (st‐ and ct‐DNA). The DNA solutions (both G4 and duplex) used for the experiments described in this figure were prepared in lithium cacodylate buffer at pH 7.3 and supplemented with K^+^ as described in the experimental details (see Supporting Information).

We were interested to study whether the emission lifetimes of the new complexes can be used as markers for binding to certain DNA topologies. Since emission lifetime is concentration‐independent, it is an attractive parameter when developing optical probes for cellular imaging. To this aim, we first recorded the time‐resolved emission decays in the absence of DNA and, subsequently, in the presence of an excess of both G4 and duplex DNA, to ensure complete binding of the probes. Complex non‐monoexponential excited state decays were observed in all cases, and therefore, we chose intensity‐weighted average lifetime (τ_w_) as a reporter. The platinum(II) complexes when free in aqueous solution displayed τ_w_ values ranging between 75 and 97 ns for the **3‐R** series, and between 147 and 269 ns for the **4‐R** compounds (see Table S4 for all the data). A large lifetime enhancement was observed upon binding to all DNA topologies. Despite small differences in binding affinities, the intensity‐weighted average lifetime (τ_w_) of the **3‐R** series (i.e. **3‐Pip**, **3‐NMe** and **3‐Mor**) upon DNA binding was found to be dependent on the topology (Figure 2), with lifetimes between 1.2 and 1.9 μs upon G4 binding compared to *ca*. 0.8 μs upon duplex binding. The **4‐R** series (**4‐Pip**, **4‐NMe** and **4‐Mor**) did not present a significant emission lifetime difference upon binding to G4 vs duplex DNA (Table S4 and Figure S49). This data indicates that the **3‐R** complexes have potential to distinguish between different DNA topologies via phosphorescence lifetime, thus, we next looked to investigate their behaviour in cells.

### Cell Viability and Fluorescence Microscopy

Prior to performing imaging studies, cell viability of osteosarcoma cell line U2OS was assessed via MTS assays by incubating the cells with 1 to 80 μM of each complex for 24 h. This data indicates that under these conditions cell viability with all **3‐R** and **4‐R** compounds remains above 95 % for concentrations below 40 μM, and is *ca*. 80 % at 80 μM (Figure S50). The treated cells were analyzed by confocal microscopy which revealed that, even at the concentration of 80 μM, there was no significant cellular uptake of any compounds, explaining the lack of cytotoxicity.

Since the probes were poorly cell‐permeable in live cells, we next set out to study their localization in fixed cells. U2OS cells were seeded and grown over 24 h, and fixed with paraformaldehyde (PFA), followed by compound addition (80 μM) and a further 24 h incubation. Cellular images revealed clear nuclear localization for the three probes (Figure S51) which, together with the clear switch‐on of their emission intensity is an indication of DNA binding. However, from confocal microscopy studies alone it is not possible to ascertain the topology of DNA they are binding to. With the aim to explore this further, we performed several visualization studies using **3‐Pip**, as the probe that showed the highest binding affinities, switch‐on effects and the longest emission lifetimes when bound to G4 s (as compared to duplex DNA).

First, we compared fluorescence microscopy images of fixed U2OS cells that were incubated with 80 μM of **3‐Pip** for 24 h, with U2OS cells that were additionally transfected with 750 ng/well of *c‐Myc* G4 DNA prior to fixation. The images obtained in the presence of additional *c‐Myc* G4 DNA revealed an increased intensity in the nuclear staining compared with un‐transfected cells (see Figures S52), suggesting that the probe preferentially binds to G4 DNA. Interestingly, the emission spectrum obtained from the cellular images—both with and without transfected G4 *c‐Myc* DNA (Figures S52c and S53)—showed a red shifted emission maxima (650 nm) compared with the in vitro spectrum of **3‐Pip** bound to DNA (580 nm) (Figure 1b).

**Figure 3:**
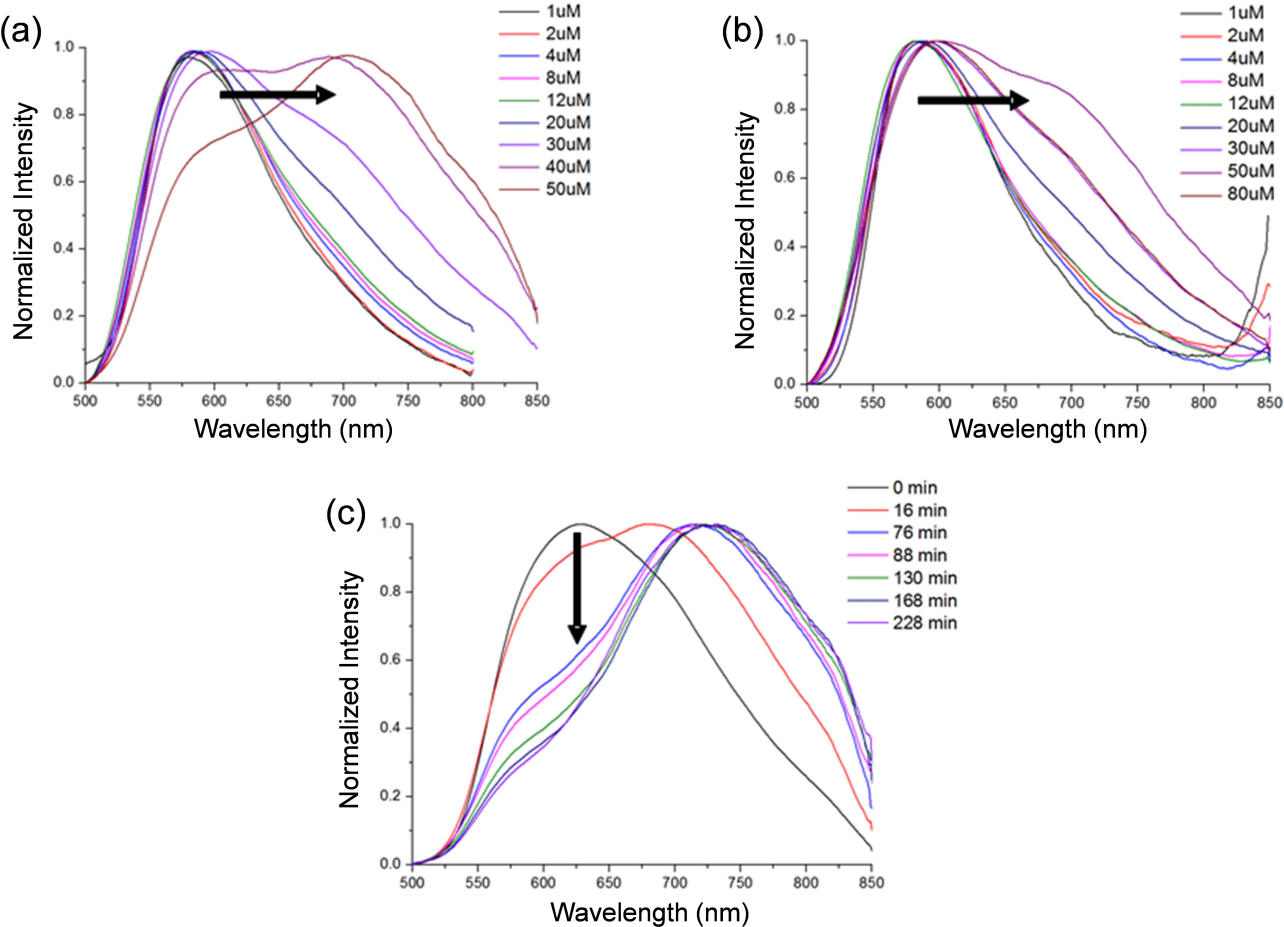
Normalized emission spectra of: a) 2 μM of *HTG4* DNA and increasing concentrations of **3‐Pip** (from 1 to 50 μM); b) 20 μM of st‐DNA and increasing concentrations of **3‐Pip** (from 1 to 80 μM); c) 2 μM of *HTG4* DNA and 50 μM of **3‐Pip** recorded over 3.5 h. The DNA solutions (both G4 and duplex) used for the titrations described in this figure were prepared in lithium cacodylate buffer at pH 7.3 and supplemented with K^+^ as described in the experimental details (see Supporting Information).

This prompted us to investigate whether the red shift could be caused by the propensity of square planar platinum(II) complexes to aggregate, leading to metal‐metal‐to‐ligand charge transfer (MMLCT) emission. Several studies have shown that platinum(II) complexes can change their luminescent properties induced by solvent, temperature, pH, and other stimuli in solution due to changes in their intermolecular π–π stacking and metal–metal interactions.[^14b^, ^21^] We therefore tested the emission properties of **3‐Pip** at high concentrations with/without DNA to identify spectroscopic features displayed by this probe upon aggregation. We first performed in vitro emission titrations where increasing amounts of **3‐Pip** were added to a constant concentration of a G4 DNA sequence (we used *HTG4* for these studies). Note, this is different to the titrations presented in Figure 1, where increasing amounts of G4 were added to a fixed concentration of the complexes. Interestingly, addition of **3‐Pip** (from 1 to 50 μM) to a 2 μM solution of *HTG4* led to a clear and progressive redshift of the emission maximum from *ca*. 580 to 710 nm (see normalized spectra in Figure 3a). The emission above 700 nm at higher concentrations of **3‐Pip** is consistent with the formation of aggregates via π–π stacking and resulting metal–metal interactions^[21]^ (we confirmed this by recording the emission spectrum of **3‐Pip** at different concentrations—see Figure S45). Interestingly, the redshifted band, while still present, was far less pronounced upon the addition of **3‐Pip** to st‐DNA (Figure 3b). We also investigated the aggregation of **3‐Pip** in the presence of DNA over time by recording the emission spectra of a solution containing *HTG4* (2 μM) and **3‐Pip** (50 μM) over 3.5 hours. The initial spectrum was broad with the maximum emission centred at *ca*. 580 nm and a shoulder at *ca*. 700 nm (Figure 3c). Over time, the peak at 700 nm became more pronounced while the one at 580 nm decreased, clearly showing that under these conditions, **3‐Pip** aggregates with time. Finally, we recorded fluorescence spectra of **3‐Pip** at 80 μM without DNA and in the presence of st‐DNA or G4 DNA using spectrally‐resolved microscopy (Figures S53 and S54), to enable direct comparison with the cellular data. We confirmed that the emission maxima at 580 nm (appearing as a shoulder) and 650 nm (main peak) are observed in microscopy images of aggregates of **3‐Pip** that form both in the absence and the presence of G4 and duplex DNA. Additionally, in the cellular data the monomer peak is more visible at lower **3‐Pip** concentrations (Figure S55). This data confirms that the red‐shifted emission (at 650 nm recorded by microscopy and 700 nm recorded using a spectrometer) belongs to the **3‐Pip** aggregates. The difference in wavelength (depending on the measurement method) for the aggregated system is due to a different detector and no sensitivity correction available in microscopy‐based spectral measurements. Taken together, the microscopy spectral data and concentration‐ and time‐dependent spectroscopy data of **3‐Pip** suggests that this compound is prone to aggregation at high concentrations, leading to the appearance of a new red‐shifted emission band and that G4 DNA promotes the aggregation of **3‐Pip**.

### Cellular detection of G4 s via PLIM

Both phosphorescence intensity and spectral data for **3‐Pip** in cells are consistent with binding to G4 DNA. To confirm that this is indeed the case, we next used phosphorescence lifetime data in the attempt to assign unambiguously the binding preference of **3‐Pip** in cells. First, we verified that the formation of aggregates does not affect the long lifetime detected for **3‐Pip** upon binding to G4 s seen in Figure 2. We recorded time‐resolved phosphorescence decays for the solutions of the free probe, the duplex‐bound probe and the G4‐bound probe in two spectral channels: 550–620 nm and 625–700 nm, corresponding to the monomer and aggregate emission, respectively (Figure S54 and Tables S5 & S6). The decay components observed in the aggregate window are short,<1 μs, irrespective of the solution studied. Hence, the presence of a long lifetime component in PLIM images of cells would confirm the detection of G4 DNA. In addition to enabling the direct detection of G4 DNA, the microsecond‐range phosphorescence lifetime displayed by our probes allows their detection over the background of autofluorescence of biological cells/tissues, as well as other fluorescent probes, characterized by lifetimes in the range of a few nanoseconds.

**Figure 4:**
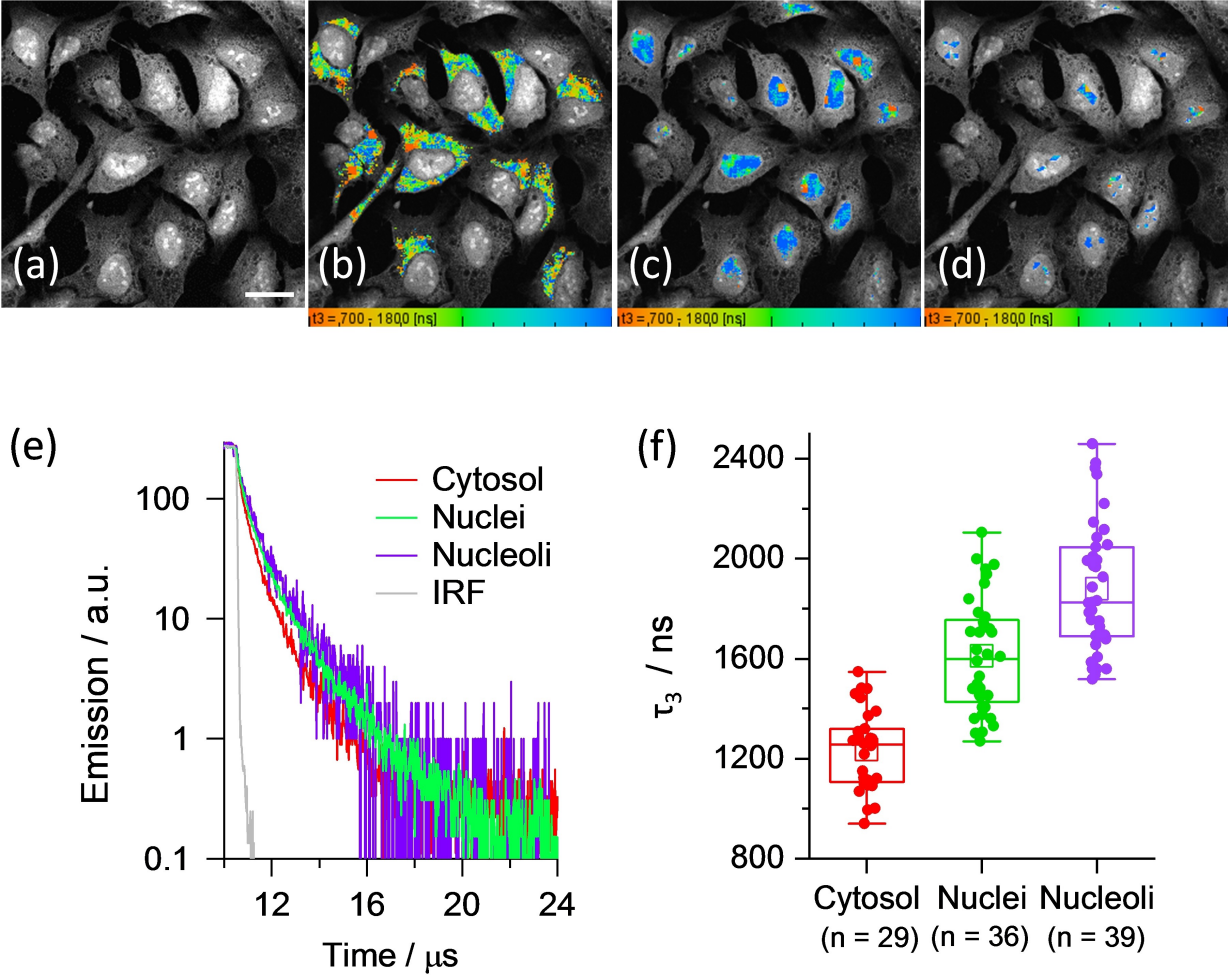
(a) Intensity and (b, c, d) PLIM images recorded for fixed U2OS cells stained with 80 μM of **3‐Pip** after 850 nm multiphoton excitation and detection in the 550–620 nm region. The segmentation of the (b) cytosol, (c) nuclei and (d) nucleoli. Scale bar is 40 μm. (e) Normalized averaged time resolved phosphorescence decays corresponding to segments shown in (b–d) and the associated statistical analysis (f), p<0.05 for all condition pairs.

PLIM images of U2OS cells incubated with 80 μM of **3‐Pip** are shown in Figure 4. The cells show intense nuclear staining, with particularly bright emission observed in the nucleoli, and a weaker cytosol staining. The segmented lifetime images of cytosol, whole nuclei and individual nucleoli are shown in Figure 4b–d, representative decay traces are shown in Figure 4e and resulting lifetimes from three‐exponential fit are listed in Table S7. The statistical data analysis for the longest component, τ_3_, is shown in Figure 4e. It is clear that the longest decay is seen in nucleoli, followed by the whole nuclei, with the shortest lifetime seen in the cytosol (Figure 4e–f). The values seen for the longest lifetime component in all cases, between 1.2–1.9 μs (Table S7, Figure 4e), indicate G4 binding of **3‐Pip**, since the weighted lifetime of the probe bound to duplex DNA is *ca*. 700 ns (Figure 2 and Table S4). The amplitude of this long lifetime component is low (*ca*. 1–2 %) which is consistent with the low abundance of G4 DNA in cells) and significant presence of aggregated **3‐Pip**. The latter could be bound to duplex or G4 DNA and is characterized by a short lifetime.

**Figure 5:**
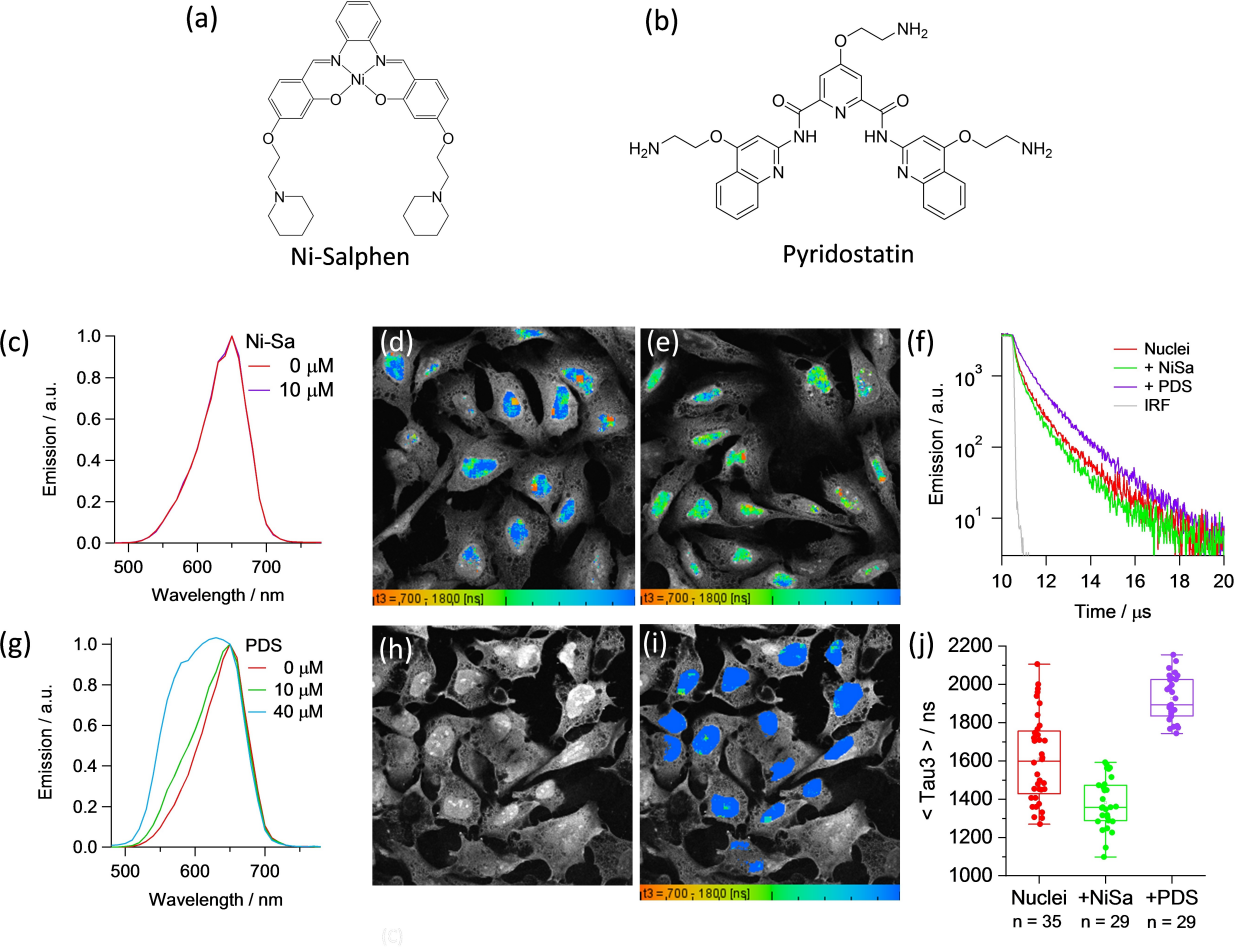
(a) Chemical structure of the G4 DNA binder, Ni‐salphen (Ni‐Sa); (b) Chemical structure of the G4 DNA binder, pyridostatin (PDS); (c) Normalized emission spectra of **3‐Pip** (80 μM) and in the absence and presence of Ni‐salphen at concentrations of 10 μM obtained from cellular images of fixed U2OS cells, excited at 850 nm; (d) PLIM images for the sample treated with **3‐Pip** (80 μM), emission detected between 550—620 nm, nuclei were selected for PLIM analysis; € PLIM images for the sample treated with **3‐Pip** (80 μM) followed by addition of Ni‐Salphen (5 μM), emission detected between —0–620 nm, nuclei were selected for PLIM analysis; (f) Normalized averaged time resolved phosphorescence decays corresponding to segmented nuclei upon addition of Ni‐Salphen and PDS; (g) Normalized emission spectra of **3‐Pip** (80 μM) and in the presence of PDS at concentrations of 10 μM (green) and 40 μM (blue) obtained from cellular images of fixed U2OS cells, excited at 850 nm; (h) emission intensity and (i) PLIM images for the sample treated with 40 μM PDS, emission detected between 550–620 nm, nuclei were selected for PLIM analysis. (j) statistical analysis of the longest lifetime component (τ_3_), recorded in control cells and in the presence of displacers, p<0.05 for all condition pairs. PLIM images visualise the distribution of τ_3_, the scale is between 700–1800 ns in all images.

We next set out to study the competitive interactions of other DNA binders in the presence of **3‐Pip**, by incubating cells with a Nickel(II)‐salphen complex (Ni‐Salph)^[22]^ and pyridostatin (PDS),^[23]^ small molecules with high affinity and selectivity for G4 s (Figure 5a–b). The lifetime analysis of the PLIM images recorded in the presence of Ni‐Salph (Figures 5c–f), shows a statistically significant shortening of the **3‐Pip** lifetime from cells, consistent with displacement of the complex by stronger binder Ni‐Salph. The spectra recorded from cells before/after incubation with this G4 binder show a mixture of monomer and aggregate bands present.

Interestingly, cells treated with **3‐Pip** and PDS presented an enhanced contribution from the monomer band at 580 nm, compared to a PDS‐free sample (Figure 5g), and **3‐Pip**’s monomer band contribution increases upon addition of increasing amounts of PDS.

The lifetime analysis of the PLIM images recorded in the presence of PDS (Figures 5i–j), also shows an increased amplitude of the longest lifetime component (1.5–2 μs) consistent with the **3‐Pip** monomer bound to G4 (see Table S8 for the data analysis and Figure S56 for additional imaging data).

We verified that the presence of PDS in vitro also causes partial disaggregation of **3‐Pip**, accompanied by an increase of the relative intensity of the 580 nm band (Figure S57). Also, an increase in the amplitude of the long lifetime component of 1.5–2 μs is observed in the presence of PDS in vitro (Figures S54, S59 and Table S5). Unusually, the addition of PDS in our experiments causes the appearance of a higher concentration of a G4‐bound probe. We hypothesize that this is due to the ability of PDS to disrupt **3‐Pip** aggregation, which was seen in vitro as well as *in cellulo*. Disruption of aggregates can be achieved by either competitive binding of PDS to some of the G4 s (thus dislodging aggregates bound to G4 s and making the monomer signal more prominent in comparison), or by directly interacting with the aggregated probe. The latter is proposed since we see a difference in emission spectra of **3‐Pip** in vitro (an increase in the monomer band), even in the absence of DNA (Figure S54). Importantly, as only G4 DNA‐bound monomers display lifetimes above 1 μs, we are confident that the presence of this long‐lived component in cells can be assigned to G4 bound **3‐Pip**.

## Conclusion

In conclusion, we present a new series of cyclometallated platinum(II) complexes that strongly interact with G‐quadruplex DNA in vitro and *in cellulo*. Upon binding to G‐quadruplex DNA, they display an increase in their phosphorescence intensity, acting as switch‐on probes, enabling their detection in fixed cells. Uniquely, upon binding to G‐quadruplex DNA they display a selective and distinct lengthening of their emission lifetime, which allowed us to directly visualise the foci of G4 DNA *in cellulo* using PLIM. These complexes also display a complex aggregation behaviour, facilitated by the presence of G4 DNA, which manifests itself in complex spectral patterns seen in vitro and *in cellulo*. However, PLIM showed itself to be a powerful methodology that allowed us to distinguish the cellular signals of G4‐bound disaggregated probes in a quantitative manner. While this allows to visualize G4 s in cells, the interplay between aggregation and DNA binding makes the interpretation of PLIM data more challenging, making it necessary to perform control experiments to avoid misinterpretation. Thus, we have performed displacement experiments with strong G4 binders and based on these results we are confident that the observed PLIM lifetime data is consistent with the presence of G4 s in cells. Overall, the considerable potential of PLIM‐based detection of G4 in cells is demonstrated here for the first time, using a new class of phosphorescent platinum(II) probes, with additional advantage of removed interference from common fluorophores displaying shorter lifetimes on the nanosecond timescale.

## Conflict of interest

The authors declare no conflict of interest.

1

## Supporting information

As a service to our authors and readers, this journal provides supporting information supplied by the authors. Such materials are peer reviewed and may be re‐organized for online delivery, but are not copy‐edited or typeset. Technical support issues arising from supporting information (other than missing files) should be addressed to the authors.

Supporting Information

## Data Availability

The data that support the findings of this study are available from the corresponding author upon reasonable request.
