# Supplementary material for: Platinum(II)‐Based Optical Probes for Imaging Quadruplex DNA Structures via Phosphorescence Lifetime Imaging Microscopy
Source: Angew Chem Int Ed Engl. 2023 Sep 8;62(42):e202310402. doi: 10.1002/anie.202310402 (PMC10952808; doi:10.1002/anie.202310402)
Supplement: Supplementary file 1 — Supporting Information [file ANIE-62-0-s001.pdf]

Angewandte Chemie
Eine Zeitschrift der Gesellschaft Deutscher Chemiker

Supporting Information

# Platinum(II)-Based Optical Probes for Imaging Quadruplex DNA Structures via Phosphorescence Lifetime Imaging Microscopy

*J. Berrones Reyes, P. S. Sherin, A. Sarkar, M. K. Kuimova*, R. Vilar**

## Supplementary Information

### Experimental Details

General Procedures. All starting materials were purchased from Sigma–Aldrich, Fluorochem or VWR and used as received from commercial sources. $^{1}$H NMR and $^{13}$C NMR spectra were recorded on a Bruker Avance 400 MHz Ultrashield NMR spectrometer and chemical shifts are reported in parts per million (ppm). Assignments were carried out with additional 2D NMR (COSY, HSQC and HMBC). Mass spectrometric analysis was performed on an Agilent 6125 MS/1260 LC by Dr. Lisa D. Haigh (Imperial College London). Elemental Analysis of the compounds prepared were performed by Dr Nigel Howard (University of Cambridge). Absorbance measurements were done using an 8453 UV/Visible Spectroscopy System (Agilent) or a Cary 60 UV/Vis Spectrometer (Agilent) and a 1 cm quartz cuvette. All fluorescence measurements were measured by using a Fluoromax-4 spectrofluorimeter (Jobin–Yvon; Horiba) using a 1 cm quartz cuvette.

### Synthesis

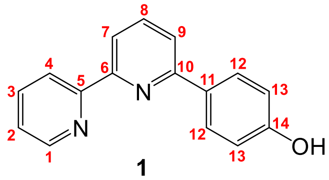


**1**

**Synthesis of 6-(4′′-hydroxy-phenyl)-2,2′-bipyridine (1).** N-[2-(2-Pyridyl)-2-oxoethyl] pyridinium iodide (3.08 g, 9.45 mmol), ammonium acetate (20.54 g, 28.2 equiv), and acetic acid (19 mL) were heated at 115 °C until everything dissolved (~10 min). Then 4′-hydroxy-3-(dimethylamino)-propiophenone hydrochloride (2.60 g, 9.64 mmol) was added, and the solution was refluxed for 6 h. The dark red-brown solution was diluted with water and neutralized with $NaHCO_3$. The solution was filtered, and the resulting red-brown residue was purified by

passing through silica gel with acetone, yielding a yellow solid. Yield: 60%. $^{1}$H NMR (400 MHz, DMSO-*d$_6$*): δ = 9.75 (s, 1H, OH), 8.65 (dd, 1H, $^{3}J_{HH}$ = 4.8 Hz, $^{4}J_{HH}$ = 1.2 Hz, H-1), 8.51 (dt, 1H, $^{3}J_{HH}$ = 8.0 Hz, $^{4}J_{HH}$ = 1.6 Hz, H-4), 8.20 (dd, 1H, $^{3}J_{HH}$ = 7.2 Hz, $^{4}J_{HH}$ = 0.8 Hz, H-7), 8.04 (d, 2H, $^{3}J_{HH}$ = 8.9 Hz, H-12), 7.93 (m, 1H, H-3), 7.90 (m, 1H, H-8), 7.84 (dd, 1H, $^{3}J_{HH}$ = 8.0 Hz, $^{4}J_{HH}$ = 1.2 Hz, H-9), 7.40 (dd, 1H, $^{3}J_{HH}$ = 7.6 Hz, 4.8, $^{4}J_{HH}$ = 1.2 Hz, H-2), 6.86 (d, 2H, $^{3}J_{HH}$ = 8.8 Hz, H-13). $^{13}$C NMR (400 MHz, DMSO-*d$_6$*): δ 158.72 (C-14), 155.57 (C- 5), 155.48 (C-10), 154.67 (C-6), 149.21 (C-1), 138.12 (C-8), 137.28 (C-3), 129.37 (C-11), 128.06 (C-12), 124.15 (C-2), 120.57 (C-4), 119.26 (C-9), 117.92 (C-7), 115.56 (C-13). TOF MS (ES$^{+}$) calculated for $C_{16}H_{12}N_2O$ 249.1033, found 249.1028.

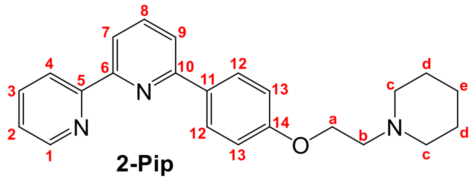

**2-Pip**

**Synthesis of ligand 2-Pip.** 6-(4′′-hydroxy-phenyl)-2,2′-bipyridine (0.248 g, 1mmol) and $K_2CO_3$ (0.276 g, 2mmol) were stirred in dry DMF (15 ml) for 10 min. Then 1-(2-Chloroethyl) piperidine hydrochloride (0.184 g, 1mmol) was added and the solution was stirred at room temperature for 72 h. The solvent was removed under vacuum, the pale-yellow residue was re-dissolved in $CH_2Cl_2$ and filtered. The solvent was removed under reduced pressure to yield the product as a pale-yellow solid. Yield: 82%. $^{1}$H NMR (400 MHz, $CDCl_3$): δ = 8.65 (dd, 1H, $^{3}J_{HH}$ = 4.8 Hz, $^{4}J_{HH}$ = 0.8 Hz, H-1), 8.59 (dt, 1H, $^{3}J_{HH}$ = 8.0 Hz, $^{4}J_{HH}$ = 1.6 Hz, H-4), 8.27 (dd, 1H, $^{3}J_{HH}$ = 8.0 Hz, $^{4}J_{HH}$ = 1.2 Hz, H-7), 8.06 (d, 2H, $^{3}J_{HH}$ = 8.8 Hz, H-12), 7.80 (m, 2H, H-3, H-8), 7.65 (d, 1H, $^{3}J_{HH}$ = 8.0 Hz, H-9), 7.26 (m, 1H, H-2), 6.99 (d, 2H, $^{3}J_{HH}$ = 8.8 Hz, H-13), 4.14 (t, 2H, $^{3}J_{HH}$ = 6.0 Hz, H-a), 2.78 (t, 2H, $^{3}J_{HH}$ = 5.6 Hz, H-b), 2.50 (m, 4H, H-c), 1.60 (p, 4H, $^{3}J_{HH}$ = 5.6 Hz, H-d), 1.43 (m, 2H, H-e). $^{13}$C NMR (400 MHz, $CDCl_3$) δ 159.81 (C-14), 156.47 (C-5), 156.07 (C- 10), 155.54 (C- 6), 149.03 (C- 1), 137.60 (C- 8), 136.82 (C- 3), 131.97 (C- 11), 128.17 (C- 12), 123.67 (C- 2), 121.27 (C-4), 119.52 (C- 9), 118.59 (C- 7), 114.77 (C- 13), 66.09 (C- a), 57.96 (C- b), 55.12 (C- c), 25.99 (C- d), 24.24 (C- e). TOF MS (ES$^{+}$) calculated for $C_{23}H_{25}N_3O$ 360.2067, found 360.2076.

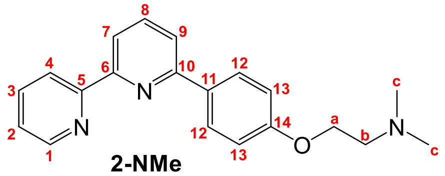

**2-NMe**

**Synthesis of ligand 2-NMe.** 6-(4′′-hydroxy-phenyl)-2,2′-bipyridine (0.248 g, 1mmol) and $K_2CO_3$ (0.276 g, 2mmol) were stirred in dry DMF (15 ml) for 10 min. Then 2-(Dimethylamino) ethyl chloride (0.144 g, 1mmol) was added, and the solution was stirred at room temperature for 72 h. The solvent was removed under vacuum, the pale-yellow residue was re-dissolved in $CH_2Cl_2$ and filtered. The solvent was removed under reduced pressure to yield the product as a pale-yellow oil. Yield: 89%. $^{1}$H NMR (400 MHz, $CDCl_3$): δ = 8.67 (dd, 1H, $^{3}J_{HH}$ = 4.8 Hz, $^{4}J_{HH}$ = 0.8 Hz, H-1), 8.60 (dt, 1H, $^{3}J_{HH}$ = 8.0 Hz, $^{4}J_{HH}$ = 1.6 Hz, H-4), 8.28 (dd, 1H, $^{3}J_{HH}$ = 8 Hz, $^{4}J_{HH}$ = 1.2 Hz, H-7), 8.08 (d, 2H, $^{3}J_{HH}$ = 8.8 Hz, H-12), 7.83 (m, 2H, H-3, H-8), 7.68 (d, 1H, $^{3}J_{HH}$ = 8.0 Hz, H-9), 7.30 (m, 1H, H-2), 7-02 (d, 2H, $^{3}J_{HH}$ = 8.8 Hz, H-13), 4.14 (t, 2H, $^{3}J_{HH}$ = 6.0 Hz, H-a), 2.77 (t, 2H, $^{3}J_{HH}$ = 5.6 Hz, H-b), 2.37 (s, 6H, H-c). $^{13}$C NMR (400 MHz, $CDCl_3$) δ 159.92 (C-14), 156.59 (C-5), 156.21 (C-10), 155.67 (C-6), 149.15 (C-1), 137.73 (C-8), 136.95 (C-3), 132.15 (C-11), 128.28 (C-12), 123.79 (C-2), 121.40 (C-4), 119.66 (C-9), 118.71 (C-7), 114.86 (C-13), 66.21 (C-a), 58.44 (C-b), 46.10 (C-c). TOF MS (ES$^{+}$) calculated for $C_{20}H_{21}N_3O$ 320.1759, found 320.1763.

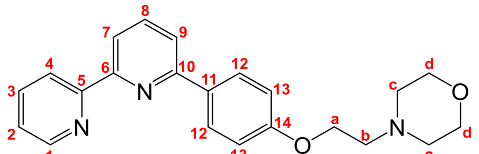

**2-Mor**

**Synthesis of ligand 2-Mor.** 6-(4′′-hydroxy-phenyl)-2,2′-bipyridine (0.248 g, 1mmol) and $K_2CO_3$ (0.276 g, 2mmol) were stirred in dry DMF (15 ml) for 10 min. Then 4-(2-Bromoethyl) morpholine hydrobromide (0.275 g, 1mmol) was added and the solution was stirred at room temperature for 72 h. The solvent was removed under vacuum, the pale-yellow residue was re-dissolved in $CH_2Cl_2$ and filtered. The solvent was removed under reduced pressure to yield the product as a pale-yellow solid. Yield: 94%. $^1$H NMR (400 MHz, $CDCl_3$): δ = 8.66 (dd, 1H, $^3J_{HH}$ = 4.8 Hz, $^4J_{HH}$ = 0.8 Hz, H-1), 8.59 (dt, 1H, $^3J_{HH}$ = 8.0 Hz, $^4J_{HH}$ = 1.6 Hz, H-4), 8.29 (dd, 1H, $^3J_{HH}$ = 8.0 Hz, $^4J_{HH}$ = 1.2 Hz, H-7), 8.07 (d, 2H, $^3J_{HH}$ = 8.8 Hz, H-12), 7.81 (m, 2H, H-3, H-8), 7.67 (d, 1H, $^3J_{HH}$ = 8.0 Hz, H-9), 7.28 (m, 1H, H-2), 7.00 (d, 2H, $^3J_{HH}$ = 8.8 Hz, H-13), 4.15 (t, 2H, $^3J_{HH}$ = 6.0 Hz, H-a), 3.73 (t, 4H, $^3J_{HH}$ = 4.8 Hz, H-d), 2.81 (t, 2H, $^3J_{HH}$ = 5.6 Hz, H-b), 2.58 (m, 4H, H-c). $^{13}$C NMR (400 MHz, $CDCl_3$) δ 159.68 (C-14), 156.48 (C-5), 156.04 (C-10), 155.60 (C-6), 149.09 (C-1), 137.67 (C-8), 136.87 (C-3), 132.20 (C-11), 128.24 (C-12), 123.73 (C-2), 121.29 (C-4), 119.58 (C-9), 118.69 (C-7), 114.79 (C-13), 66.99 (C-a), 65.93 (C-d), 57.69 (C-b), 54.18 (C-d). TOF MS (ES$^+$) calculated for $C_{22}H_{23}N_3O$ 362.1874, found 362.1869.

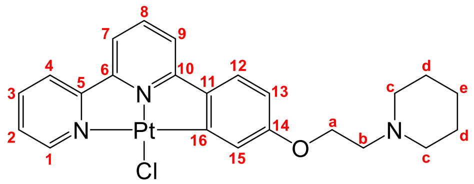

**3-Pip**

**Synthesis of compound 3-Pip.** Compound **2-Pip** (0.100 g, 0.28 mmol) and sodium acetate (0.022 g, 0.28 mmol) were stirred in acetonitrile (15 ml) for 10 min. $K_2PtCl_4$ (0.116 g, 0.28 mmol) and DMSO (0.5 ml) were then added, and the reaction mixture was stirred at 65 °C for 72 h while a yellow precipitate formed. The volume of the solution was reduced to about 5 ml and pentane (20 ml) was added. The resulting precipitate was filtered off, washed with water (30 ml), pentane (15 ml), diethyl ether (15 ml) and dried in vacuo. The solid was re-dissolved in DCM, filtered off and the volume of the filtrate was reduced to about 5 ml. Addition of pentane afforded a pure compound as a yellow solid. Yield: 91%. $^1$H NMR (400 MHz, DMSO-$d_6$): δ = 8.86 (d, 1H, $^3J_{HH}$ = 4.8 Hz, H-1), 8.45 (d, 1H, $^3J_{HH}$ = 8.0 Hz, H-4), 8.31 (dt, 1H, $^3J_{HH}$ = 8.0 Hz, $^4J_{HH}$ = 1.2 Hz, H-3), 8.04 (m, 2H, H-8, H-9), 7.88 (t, 1H, $^3J_{HH}$ = 6.0 Hz, H-2), 7.80 (dd, 1H, $^3J_{HH}$ = 7.2 Hz, $^4J_{HH}$ = 1.6 Hz, H-7), 7.54 (d, 1H, $^3J_{HH}$ = 8.4 Hz, H-12), 6.98 (d, 1H, $^4J_{HH}$ = 2.8 Hz, $^3J_{HPt}$ = 45.6 Hz, H-15), 6.29 (dd, 1H, $^3J_{HH}$ = 8.8 Hz, $^4J_{HH}$ = 2.8 Hz, H-13), 4.05 (t, 2H, $^3J_{HH}$ = 5.6 Hz, H-a), 2.65 (t, 2H, $^3J_{HH}$ = 6.0 Hz, H-b), 2.43 (m, 4H, H-c), 1.50 (p, 4H, $^3J_{HH}$ = 5.6 Hz, H-d), 1.37 (m, 2H, H-e). $^{13}$C NMR (400 MHz, DMSO-$d_6$) δ 165.25 (C-10), 160.11 (C-16), 157.04 (C-5), 154.22 (C-6), 148.44 (C-1), 144.82 (C-14), 140.78 (C-3), 139.70 (C-8), 139.40 (C-11), 128.36 (C-2), 126.92 (C-12), 124.05 (C-4), 119.95 (C-15), 118.89 (C-7), 118.42 (C-9), 109.78 (C-13), 65.55 (C-a), 57.61 (C-b), 54.68 (C-c), 25.83 (C-d), 24.18 (C-e). TOF MS (ES$^+$) calculated for $C_{23}H_{24}ClN_3OPt$ 590.1342, found 590.1336. Elemental analysis calcd for $C_{23}H_{24}ClN_3OPt \cdot 0.25H_2O$: C 46.55, H 4.16, N 7.08; found: C 46.53, H 3.69, N 6.90.

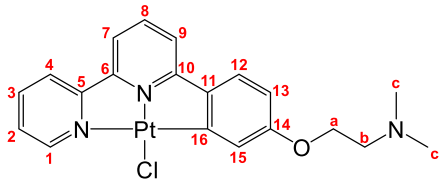

**3-NMe**

**Synthesis of compound 3-NMe.** Compound **2-NMe** (0.100 g, 0.31 mmol) and sodium acetate (0.025 g, 0.31 mmol) were stirred in acetonitrile (15 ml) for 10 min. $K_2PtCl_4$ (0.129 g, 0.31 mmol) and DMSO (0.5 ml) were then added, and the reaction mixture was stirred at 65 °C for 72 h while a yellow precipitate formed. The volume of the solution was reduced to about 5 ml and pentane (20 ml) was added. The resulting precipitate was filtered off, washed with water (30 ml), pentane (15 ml), diethyl ether (15 ml) and dried in vacuo. The solid was re-dissolved in DCM, filtered off and the volume of the filtrate was reduced to about 5 ml. Addition of pentane afforded a pure compound as a yellow solid. Yield: 96%. $^1H$ NMR (400 MHz, DMSO-$d_6$): δ = 8.84 (d, 1H, $^3J_{HH}$ = 4.0 Hz, H-1), 8.46 (d, 1H, $^3J_{HH}$ = 7.6 Hz, H-4), 8.29 (dt, 1H, $^3J_{HH}$ = 7.6 Hz, $^4J_{HH}$ = 1.2 Hz, H-3), 8.05 (m, 2H, H-8, H-9), 7.86 (t, 1H, $^3J_{HH}$ = 6.4 Hz, H-2), 7.78 (dd, 1H, $^3J_{HH}$ = 7.6 Hz, $^4J_{HH}$ = 1.6 Hz, H-7), 7.51 (d, 1H, $^3J_{HH}$ = 8.8 Hz, H-12), 6.97 (d, 1H, $^4J_{HH}$ = 2.4 Hz, $^3J_{HPt}$ = 50.0 Hz, H-15), 6.60 (dd, 1H, $^3J_{HH}$ = 8.8 Hz, $^4J_{HH}$ = 2.8 Hz, H-13), 4.04 (t, 2H, $^3J_{HH}$ = 5.6, H-a), 2.62 (t, 2H, $^3J_{HH}$ = 6, H-b), 2.22 (s, 4H, H-c). $^{13}C$ NMR (400 MHz, DMSO-$d_6$) δ 165.00 (C-10), 159.87 (C-16), 156.82 (C-5), 153.99 (C-6), 148.19 (C-1), 144.65 (C-14), 140.53 (C-3), 139.46 (C-8), 139.21 (C-11), 128.12 (C-2), 126.69 (C-12), 123.85 (C-4), 119.67 (C-15), 118.67 (C-7), 118.23 (C-9), 109.58 (C-13), 65.54 (C-a), 57.72 (C-b), 45.63 (C-c). TOF MS (ES$^+$) calculated for $C_{20}H_{20}ClN_3OPt$ 550.1002, found 550.1023. Elemental analysis calcd for $C_{20}H_{20}ClN_3OPt \cdot 0.33CHCl_3$: C 41.50, H 3.48, N 7.14; found: C 41.41, H 3.51, N 6.90.

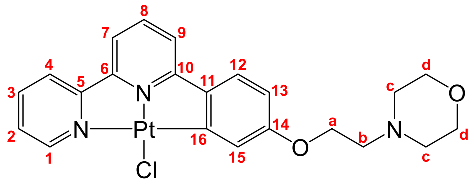

**3-Mor**

**Synthesis of compound 3-Mor.** Compound **2-Mor** (0.100 g, 0.28 mmol) and sodium acetate (0.022 g, 0.28 mmol) were stirred in acetonitrile (15 ml) for 10 min. $K_2PtCl_4$ (0.116 g, 0.28 mmol) and DMSO (0.5 ml) were then added, and the reaction mixture was stirred at 65 °C for 72 h while a yellow precipitate formed. The volume of the solution was reduced to about 5 ml and pentane (20 ml) was added. The resulting precipitate was filtered off, washed with water (30 ml), pentane (15 ml), diethyl ether (15 ml) and dried in vacuo. The solid was re-dissolved in DCM, filtered off and the volume of the filtrate was reduced to about 5 ml. Addition of pentane afforded a pure compound as a red solid. Yield: 98%. $^1H$ NMR (400 MHz, DMSO-$d_6$): δ = 8.85 (d, 1H, $^3J_{HH}$ = 4.4 Hz, H-1), 8.47 (d, 1H, $^3J_{HH}$ = 8.0 Hz, H-4), 8.31 (dt, 1H, $^3J_{HH}$ = 7.6 Hz, $^4J_{HH}$ = 1.6 Hz, H-3), 8.04 (m, 2H, H-8, H-9), 7.88 (t, 1H, $^3J_{HH}$ = 6.4 Hz, H-2), 7.80 (dd, 1H, $^3J_{HH}$ = 6.8 Hz, $^4J_{HH}$ = 0.8 Hz, H-7), 7.53 (d, 1H, $^3J_{HH}$ = 8.8 Hz, H-12), 6.99 (d, 1H, $^4J_{HH}$ = 2.8 Hz, $^3J_{HPt}$ = 46.0 Hz, H-15), 6.63 (dd, 1H, $^3J_{HH}$ = 8.4 Hz, $^4J_{HH}$ = 2.4 Hz, H-13), 4.07 (t, 2H, $^3J_{HH}$ = 5.6 Hz, H-a), 3.56 (m, 4H, H-d), 2.65 (t, 2H, $^3J_{HH}$ = 6.0 Hz, H-b), 2.47 (m, 4H, H-c). $^{13}C$ NMR (400 MHz, DMSO-$d_6$) δ 165.03 (C-10), 159.84 (C-16), 156.83 (C-5), 154.03 (C-6), 148.24 (C-1), 144.61 (C-14), 140.59 (C-3), 139.52 (C-8), 139.25 (C-11), 128.17 (C-2), 126.71 (C-12), 123.84 (C-4), 119.78 (C-15), 118.71 (C-7), 118.24 (C-9), 109.57 (C-13), 66.18 (C-a), 65.08 (C-d), 57.04 (C-b), 53.67 (C-c). TOF MS (ES$^+$) calculated for $C_{22}H_{22}ClN_3O_2Pt$ 592.1111, found 592.1128. Elemental analysis calcd for $C_{22}H_{22}ClN_3O_2Pt \cdot CHCl_3$: C 38.89, H 3.26, N 5.92; found: C 38.87, H 3.10, N 5.98.

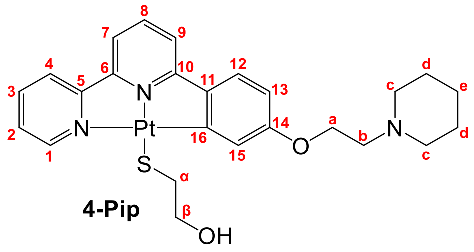


**Synthesis of compound 4-Pip.** Compound **3-Pip** (0.100 g, 0.17 mmol) and sodium methoxide (0.010 g, 0.17 mmol) were stirred in methanol (15 ml) for 10 min. Mercaptoethanol (0.013 g, 0.17 mmol) was then added and the reaction mixture was stirred under nitrogen at 60 °C for 6 h. The solvent was removed under vacuum, and the resulting residue was extracted with DCM (20 ml x 3), washed thoroughly with water (30 ml x 5) and dried over sodium sulphate. The solvent was removed under reduced pressure to yield the product as a purple solid. Yield: 48%. $^1$H NMR (400 MHz, $CDCl_3$): δ = 8.97 (d, 1H, $^3J_{HH}$ = 4.8 Hz, H-1), 7.84 (t, 1H, $^3J_{HH}$ = 7.6 Hz, H-3), 7.72 (d, 1H, $^3J_{HH}$ = 8.0 Hz, H-4), 7.55 (t, 1H, $^3J_{HH}$ = 7.6 Hz, H-8), 7.38 (m, 2H, H-2, H-7), 7.30 (d, 1H, $^3J_{HH}$ = 2.4 Hz, H-15), 7.15 (d, 1H, $^3J_{HH}$ = 8.0 Hz, H-9), 7.11 (d, 1H, $^3J_{HH}$ = 8.8 Hz, H-12), 6.52 (dd, 1H, $^3J_{HH}$ = 8.4 Hz, $^4J_{HH}$ = 2.4 Hz, H-13), 4.19 (t, 2H, $^3J_{HH}$ = 6.0 Hz, H-a), 3.72 (t, 2H, $^3J_{HH}$ = 5.6 Hz, H-β), 2.99 (t, 2H, $^3J_{HH}$ = 5.6 Hz, $^3J_{HPt}$ = 60.0 Hz, H-α), 2.77 (t, 2H, $^3J_{HH}$ = 6.0 Hz, H-b), 2.52 (m, 4H, H-c), 1.61 (p, 4H, $^3J_{HH}$ = 5.6 Hz, H-d), 1.44 (q, 2H, $^3J_{HH}$ = 4.8 Hz, H-e). $^{13}$C NMR (400 MHz, $CDCl_3$) δ 164.65 (C-10), 161.25 (C-16), 158.82 (C-5), 153.44 (C-6), 149.03 (C-1), 145.51 (C-14), 140.02 (C-11), 139.32 (C-3), 138.56 (C-8), 127.12 (C-2), 126.57 (C-12), 123.02 (C-4), 120.94 (C-15), 118.35 (C-9), 117.17 (C-7), 111.21 (C-13), 66.21 (C-a), 65.36 (C-β), 58.36 (C-b), 55.54 (C-c), 34.07 (C-α), 26.37 (C-d), 24.66 (C-c). TOF MS (ES$^+$) calculated for $C_{25}H_{29}N_3O_2PtS$ 631.1692, found 631.1707. Elemental analysis calcd for $C_{25}H_{29}N_3O_2PtS\cdot0.5H_2O$: C 44.37, H 4.31, N 6.09; found: C 44.21, H 4.30, N 5.79.

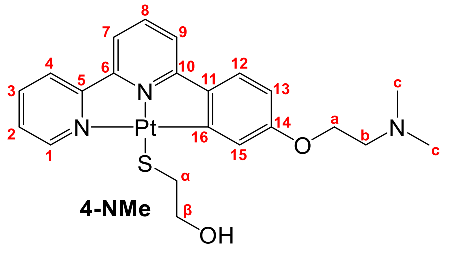


**Synthesis of compound 4-NMe.** Compound **3-NMe** (0.100 g, 0.18 mmol) and sodium methoxide (0.010 g, 0.18 mmol) were stirred in methanol (15 ml) for 10 min. Mercaptoethanol (0.014 g, 0.18 mmol) was then added and the reaction mixture was stirred under nitrogen at 60 °C for 6 h. The solvent was removed under vacuum, and the resulting residue was extracted with DCM (20 ml x 3), washed thoroughly with water (30 ml x 5) and dried over sodium sulphate. The solvent was removed under reduced pressure to yield the product as a purple solid. Yield: 33%. $^1$H NMR (400 MHz, $CDCl_3$): δ = 8.99 (d, 1H, $^3J_{HH}$ = 4.8 Hz, H-1), 7.86 (t, 1H, $^3J_{HH}$ = 7.6 Hz, H-3), 7.74 (d, 1H, $^3J_{HH}$ = 8.0 Hz, H-4), 7.57 (t, 1H, $^3J_{HH}$ = 7.6 Hz, H-8), 7.40 (m, 2H, H-2, H-7), 7.33 (d, 1H, $^3J_{HH}$ = 2.4 Hz, H-15), 7.17 (d, 1H, $^3J_{HH}$ = 8.0 Hz, H-9), 7.13 (d, 1H, $^3J_{HH}$ = 8.8 Hz, H-12), 6.59 (dd, 1H, $^3J_{HH}$ = 8.4 Hz, $^4J_{HH}$ = 2.4 Hz, H-13), 4.15 (t, 2H, $^3J_{HH}$ = 6.0 Hz, H-a), 3.72 (t, 2H, $^3J_{HH}$ = 5.6 Hz, H-β), 3.00 (t, 2H, $^3J_{HH}$ = 5.6 Hz, $^3J_{HPt}$ = 60.4 Hz, H-α), 2.74 (t, 2H, $^3J_{HH}$ = 6.0 Hz, H-b), 2.35 (s, 6H, H-c). $^{13}$C NMR (400 MHz, $CDCl_3$) δ 164.39 (C-10), 160.90 (C-16), 158.42 (C-5), 153.06 (C-6), 148.72 (C-1), 145.09 (C-14), 139.55 (C-11), 138.92 (C-3), 138.17 (C-8), 126.77 (C-2), 126.17 (C-12), 122.51 (C-4), 120.20 (C-15), 117.95 (C-9), 116.62 (C-7), 111.19 (C-13), 65.86 (C-a), 64.91 (C-β), 58.29 (C-b), 45.96 (C-c), 33.70 (C-α). TOF MS (ES$^+$) calculated for $C_{22}H_{25}N_3O_2PtS$ 591.1402, found 591.1394. Elemental analysis calcd for $C_{22}H_{25}N_3O_2PtS\cdot0.25\ CHCl_3$: C 43.07, H 4.10, N 6.77; found: C 43.01, H 4.05, N 6.21.

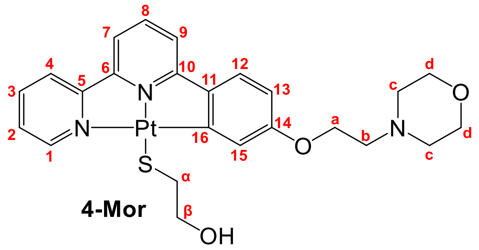

4-Mor

**Synthesis of compound 4-Mor.** Compound **3-Mor** (0.100 g, 0.17 mmol) and sodium methoxide (0.010 g, 0.17 mmol) were stirred in methanol (15 ml) for 10 min. Mercaptoethanol (0.013 g, 0.17 mmol) was then added and the reaction mixture was stirred under nitrogen at 60 °C for 6 h. The solvent was removed under vacuum, and the resulting residue was extracted with DCM (20 ml x 3), washed thoroughly with water (30 ml x 5) and dried over sodium sulphate. The solvent was removed under reduced pressure to yield the product as a purple solid. Yield: 57%. $^{1}$H NMR (400 MHz, $CDCl_3$): δ = 8.97 (d, 1H, $^{3}J_{HH}$ = 4.8 Hz, H-1), 7.84 (t, 1H, $^{3}J_{HH}$ = 7.6 Hz, H-3), 7.72 (d, 1H, $^{3}J_{HH}$ = 8.0 Hz, H-4), 7.55 (t, 1H, $^{3}J_{HH}$ = 7.6 Hz, H-8), 7.38 (m, 2H, H-2 Hz, H-7), 7.30 (d, 1H, $^{3}J_{HH}$ = 2.4 Hz, H-15), 7.15 (d, 1H, $^{3}J_{HH}$ = 8.0 Hz, H-9), 7.11 (d, 1H, $^{3}J_{HH}$ = 8.8 Hz, H-12), 6.52 (dd, 1H, $^{3}J_{HH}$ = 8.4 Hz, $^{4}J_{HH}$ = 2.4 Hz, H-13), 4.19 (t, 2H, $^{3}J_{HH}$ = 6.0 Hz, H-a), 3.72 (m, 6H, H-d, H-β), 2.99 (t, 2H, $^{3}J_{HH}$ = 5.6 Hz, $^{3}J_{HPt}$ =60.0 Hz, H-α), 2.81 (t, 2H, $^{3}J_{HH}$ = 6.0 Hz, H-b), 2.59 (t, 4H, $^{3}J_{HH}$ = 4.4 Hz, H-c). $^{13}$C NMR (400 MHz, $CDCl_3$) δ 164.02 (C-10), 160 (C-16).67, 158.31 (C-5), 152.97 (C-6), 148.56 (C-1), 145.22 (C-14), 139.80 (C-11), 138.97 (C-3), 138.26 (C-8), 126.71 (C-2), 126.17 (C-12), 122.84 (C-4), 120.39 (C-15), 117.94 (C-9), 117.07 (C-7), 110.79 (C-13), 67.03 (C-d), 65.70 (C-a), 64.93 (C-β), 57.76 (C-b), 54.25 (C-c), 33.68 (C-α). TOF MS ($ES^+$) calculated for $C_{24}H_{27}N_3O_3PtS$ 633.1511, found 633.1499. Elemental analysis calcd for $C_{24}H_{27}N_3O_3PtS \cdot CHCl_3$ : C 39.93, H 3.75, N 5.59; found: C 40.73, H 3.79, N 5.74.

## Oligonucleotide Preparation

The oligonucleotides (see below for sequences) were purchased from Eurogentec. Calf thymus DNA (ct-DNA) and salmon testes (st-DNA) were purchased from Sigma Aldrich. Stock solutions were prepared via dilution in 10 mM lithium cacodylate buffer at pH 7.3 and the concentrations were determined by UV-Vis titrations, using the molar extinction coefficients: 13200 $M^{-1}$ $cm^{-1}$ (base pair for ct-DNA and st-DNA), 228500 $M^{-1}$ $cm^{-1}$ (strand for HTelo), 228700 $M^{-1}$ $cm^{-1}$ (strand for cMyc), 205600 $M^{-1}$ $cm^{-1}$ (strand for c-kit2) and 244300 $M^{-1}$ $cm^{-1}$ (strand for HTG4). Concentrations of G4 and ds-DNA are expressed as per strand, and per base pair, respectively. Prior to use, the appropriate salt was added to a final concentration of 100 mM and the G4 DNA stock solutions were annealed by heating at 95 °C for 5 minutes and then cooling to room temperature overnight.

| Oligonucleotide | Sequence (5' to 3') |
|---|---|
| **HTelo** | AGGGTTAGGGTTAGGGTTAGGG |
| **cMyc** | TGAGGGTGGGTAGGGTGGGTAA |
| **c-kit2** | CGGGCGGGCGCGAGGGAGGGG |
| **HTG4** | TTGGGTTAGGGTTAGGGTTAGGGA |

## Fluorescence titrations

The emission spectra were recorded on a Fluoromax-4 spectrofluorimeter. Sample preparation was carried out in the appropriate buffer using a constant concentration of the probe (1 µM) , and 0-12 equivalents of HTelo ($Na^+$) (100 mM NaCl,10 mM Li cacodylate, pH7.3), HTelo ($K^+$) (100 mM KCl,10 mM Li cacodylate, pH7.3), cMyc (100 mM KCl,10 mM Li cacodylate, pH7.3), c-kit2 (100 mM KCl,10 mM Li cacodylate, pH7.3) and HTG4 (100 mM

KCl, 10 mM Li cacodylate, pH7.3) were used, and 0-180 equivalents for ct-DNA (100 mM KCl,10 mM Li cacodylate, pH7.3) and st-DNA (100 mM KCl,10 mM Li cacodylate, pH7.3). Excitation was carried out at 350 nm and the spectra were recorded from 515-680 nm. The emission spectra were performed after each aliquot addition of the DNA until saturation in fluorescence was reached. A 1 cm path-length quartz cuvette was used to carry out the measurements. Experiments were conducted in triplicate and binding constants were obtained by curve fitting in Origin Pro using the equation below.

$$y = 0.5R[1/K + L + nx - [(1/K + L + nx)^2 - 4Lnx]^{0.5}$$

Fluorescence response (y) was fitted against DNA concentration (x); R is the machine response, K is the association constant of ligand and DNA, L is the concentration of ligand and n is the number of binding sites per DNA (2).

**CD melting experiments**

CD experiments were conducted using a Jasco J-715 CD spectrometer scanning from 320 to 220 nm with a pitch of 0.1 nm, at 100 nm min$^{-1}$. The signal at 265 and 295 nm for cMyc and HTelo $Na^+$ respectively was monitored as temperature was increased from 20-95 °C at a rate of °C /min. Experiments were conducted in triplicate using a 1 cm quartz cuvette and all analysis was carried out using Origin Pro. Samples were prepared using 5 µM oligonucleotide, annealed in 10 mM LiCaco buffer supplemented with either 1mM KCl or 100 mM NaCl for cMyc and HTelo $Na^+$ respectively. Samples were subsequently mixed with 10 µM of ligand. Melting temperature ($T_m$) was determined by normalizing the ellipticity (*mdeg*) between 0 – 1 and taking the temperature at which the normalized *mdeg* was 0.5.

**In vitro time-correlated single photon counting (TCSPC)**

Time-resolved fluorescence decays were acquired using an IBH 5000F (Jobin Ybon, Horiba) time-correlated single photon counting (TCSPC) device equipped with a 404 nm NanoLED as an excitation source (pulse width < 200 ps, HORIBA) with a 13 µs time window and 4096 time bins. Decays were detected at λem = 580 nm (± 32 nm) after passing through a 495 nm long pass filter to remove any scattered excitation pulse. A neutral density filter was used for the instrument response function (IRF) measurements using a Ludox solution, detecting the emission at the excitation wavelength. Decay traces were fitted by iterative reconvolution to the equation $I(t) = I_0\left(a_1\, e^{-t/\tau 1} + a_2\, e^{-t/\tau 2}\right)$ where α1 and α2 are variables normalized to unity. The intensity-weighted average lifetime ($\tau_w$) was calculated using the equation:

$$\tau_w = \frac{\tau_1^2\,\alpha_1 + \tau_2^2\,\alpha_2}{\tau_1\,\alpha_1 + \tau_2\,\alpha_2}$$

A prompt shift was included in the fitting to consider differences in the emission wavelength between the IRF and measured decay. The goodness of fit was judged by consideration of the deviations from the model via a weighted residuals plot.

**General Details for cell culture**

Human Bone Osteosarcoma Epithelial Cells (U2OS, from ATCC) were grown in high glucose Dulbecco's modified Eagle medium (DMEM) containing 10% fetal bovine serum at 37 °C with 5% $CO_2$ in humidified air.

**Cell Viability**

MTS Reagent Powder (Promega) was dissolved in sterile PBS as a 2 mg/mL solution. Phenazine methosulfate (PMS, Sigma Life Science) was dissolved in PBS as a 0.92 mg/mL solution. On the day of experiment, 3 mL of MTS solution was mixed with 150 µL of PMS and 3 mL of the resulting solution was mixed with 15 mL of media. U2OS cells were seeded (5000 cells, 200 µL) in a 96-well plate in a DMEM medium containing 10% FBS. After 24 h the culture media was removed, and fresh media added with compounds at required concentrations (80, 40, 20, 10, 5 and 1 µM) in triplicate. Wells were maintained without compound (only cells and DMSO) and without cells (only culture medium) as 100 % and 0 % viability controls, respectively. After further 24h, the medium was replaced with medium containing MTS/PMS (100 µL). After 4 h, absorbance at 490 nm (MTS) and 635 nm (background) was measured. Cell viability was calculated from the dose response curve of absorbance (MTS – background).

**Confocal imaging**

Emission images were collected using an inverted confocal laser scanning microscope (Leica SP5 II). Pt (II) bipyridine emission (540 - 640 nm) was collected following 458 nm excitation. A 100x (HCX PL APO CS, N. A. = 1.0, oil immersion, Leica) objective was used to collect images at 512 x 512 pixel resolution.

**Fixed Cell experiments**

Cells were seeded on chambered coverglass ($1.5 \times 10^4$ cells, 250 µL) and incubated for 24 h. Cells were washed (x3) in ice-cold PBS before incubation with ice-cold paraformaldehyde (PFA, 4% in PBS) solution for 10 min, and a further wash (x3) with ice-cold PBS. Fixed cells were further treated with the compounds under study (80 µM, 250 µL) and visualized under the microscope after 3-24 h.

**Phosphorescence Lifetime Imaging Microscopy (PLIM)**

PLIM imaging was performed using a Leica SP5-II microscope coupled with a SPC-830 photon counting card (Becker & Hickl). Two-photon excitation from a Coherent Chameleon Ti:Sapphire laser was used at 850 nm. A 100× objective (HCX PL APO CS, N. A. = 1.0, oil immersion, Leica) was used. The fluorescence was detected over two ranges of 550-620 nm and 625-700 nm using two detectors. The resolution of FLIM images was 256×256 pixels with 256 channels. The IRF was obtained by measuring the second harmonic generation (SHG) signal from crystals of urea. The FLIM data were analyzed using the SPCImage software (Becker & Hickl) using triexponential decay model.

**G4 sequence transfection**

Twenty-four hours before transfection, cells ($2.5 \times 10^4$) were seeded on a chambered coverglass. cMyc G4 sequence (750 ng/ well) was transfected using 1 µL Lipofectamine 2000 (Invitrogen) per well, according to manufacturer's protocol. After a further 24 h, cells were fixed (PFA, 4% in PBS) and the probe was added (80 µM).

## Displacement assay

Fixed cells were treated with either pyridostatin (PDS), a selective G4 binder with a greater affinity for G4. After 2 h cells were exposed to the Pt bipyridines complexes and cells were observed under the microscope after 2 h.

## Synthesis and characterization

### *Synthesis*

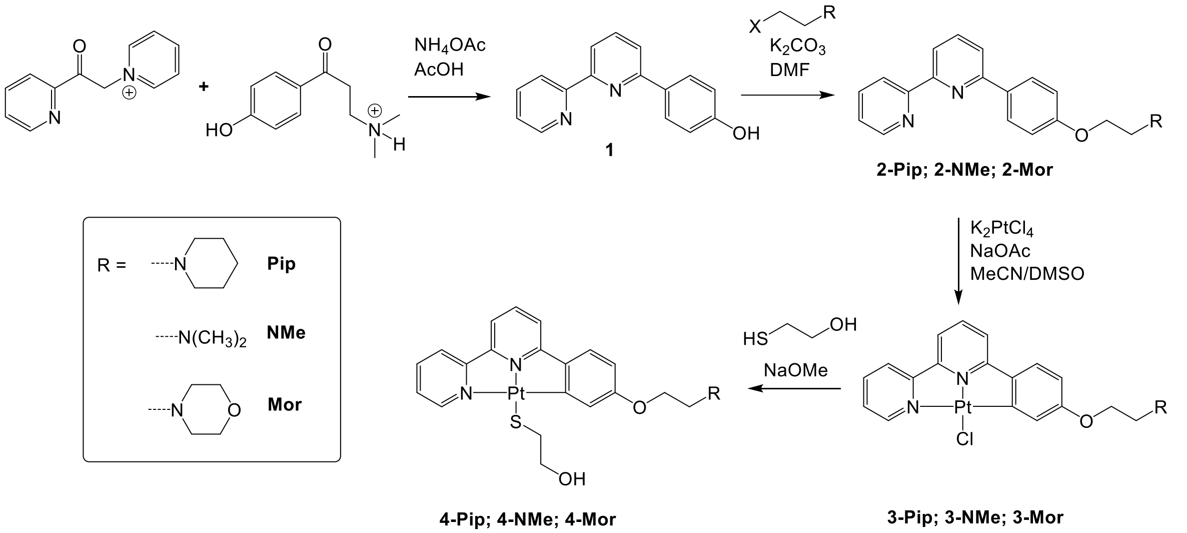


**Scheme 1.** Synthesis of bipyridine-based ligands and the corresponding cyclometallated platinum (II) complexes.

*Characterization*

- NMR spectra of ligands **2-Pip**, **2-NMe** and **2-Mor**.

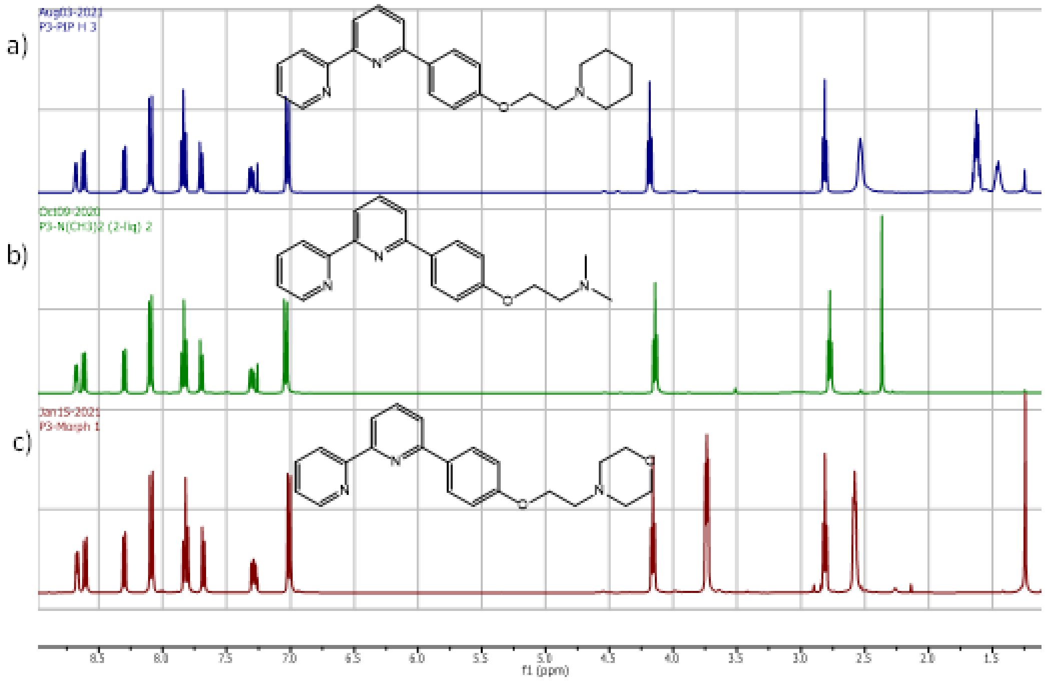

**Figure S1.** $^{1}$H NMR spectra of a) **2-Pip**, b) **2-NMe** and c) **2-Mor**.

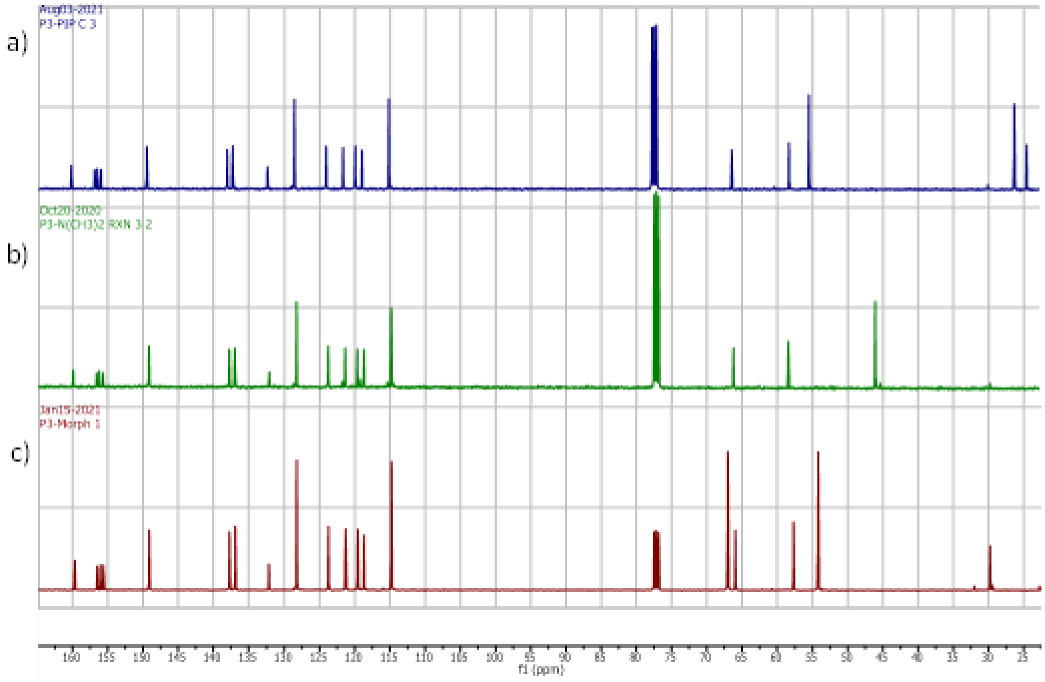

**Figure S2.** $^{13}$C NMR spectra of a) **2-Pip**, b) **2-NMe** and c) **2-Mor**.

- NMR spectra of complexes **3-Pip**, **3-NMe** and 3-**Mor**.

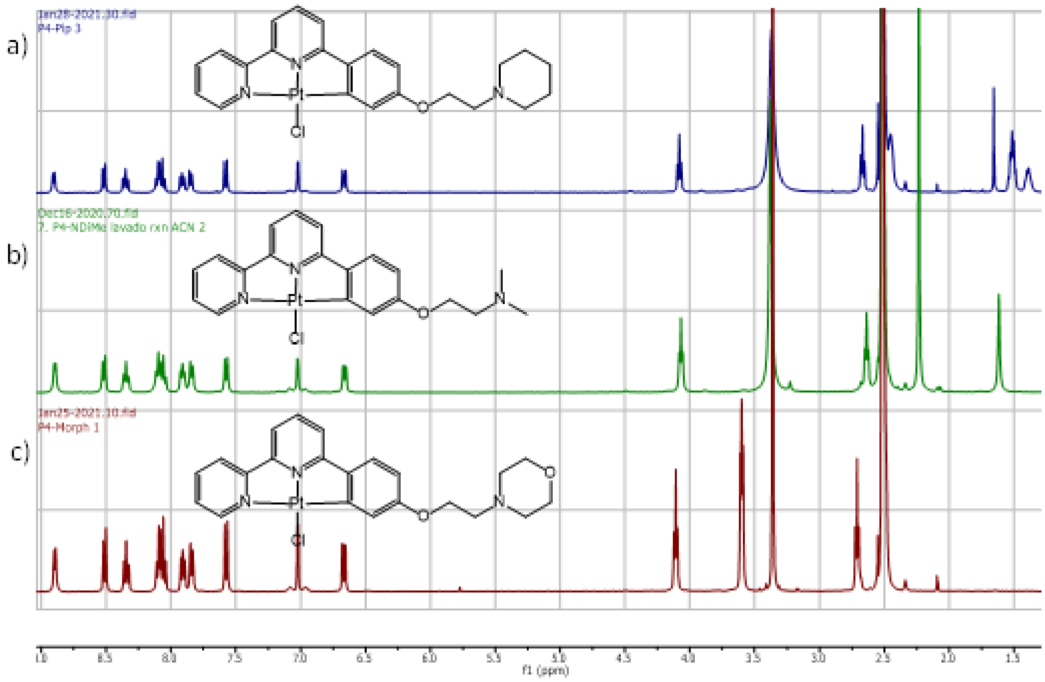


**Figure S3.** $^{1}$H NMR spectra of a) **3-Pip**, b) **3-NMe** and c) **3-Mor**.

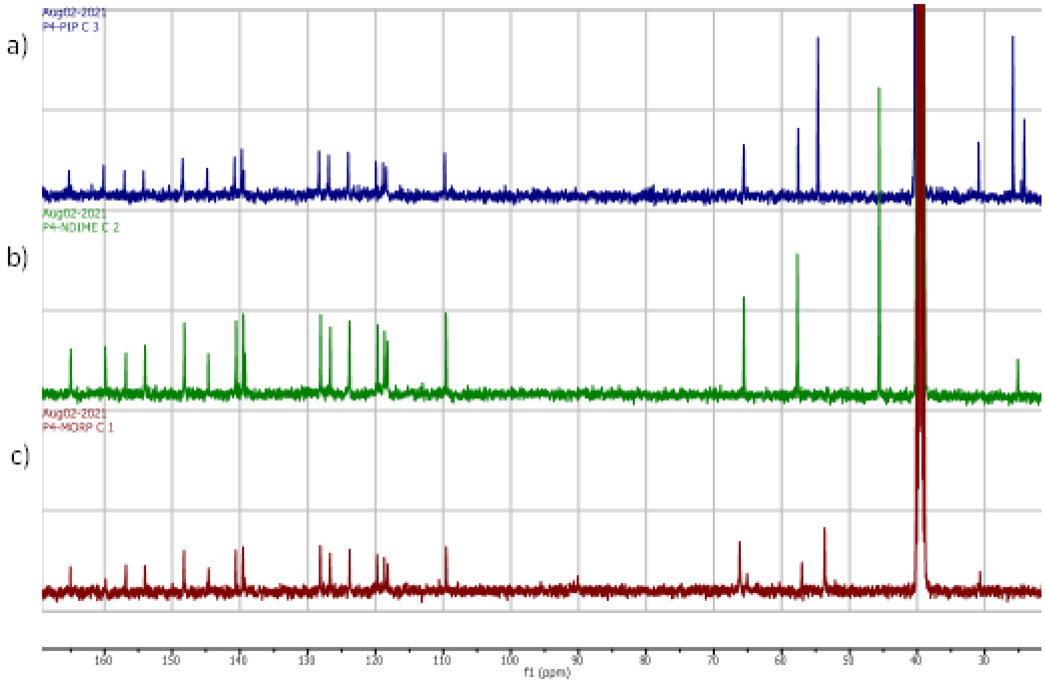


**Figure S4**. $^{13}$C NMR spectra of a) **3-Pip**, b) **3-NMe** and c) **3-Mor**.

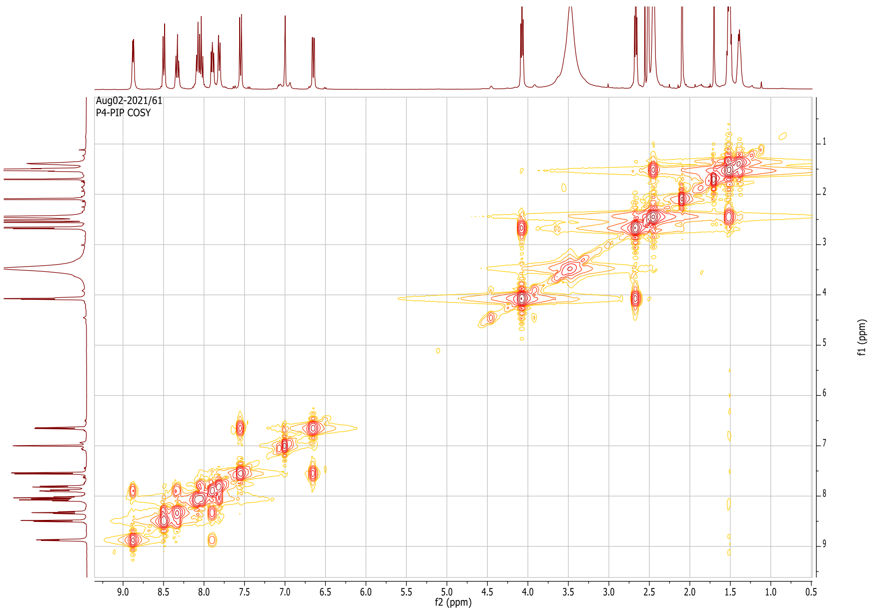


**Figure S5.** COSY NMR spectra of **3-Pip**.

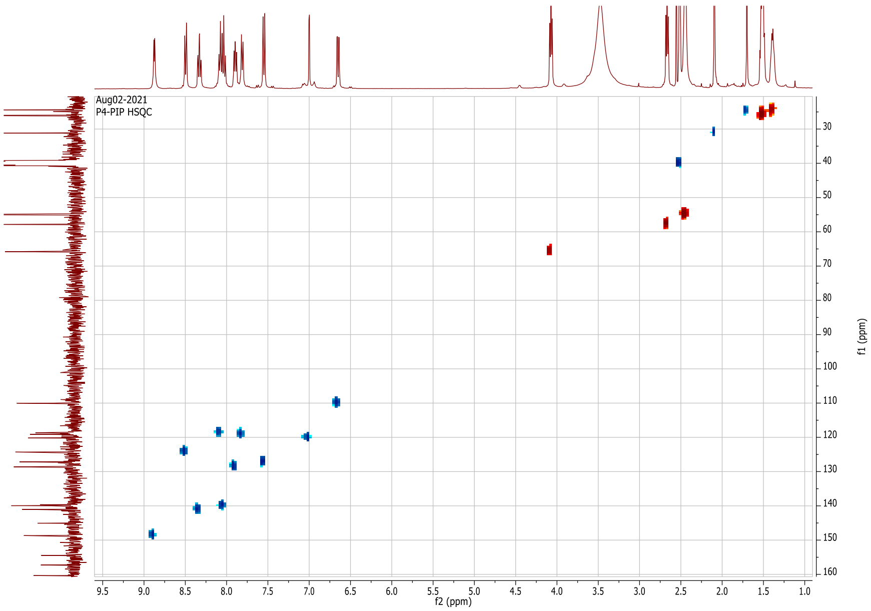


**Figure S6.** HSQC NMR spectra of **3-Pip**.

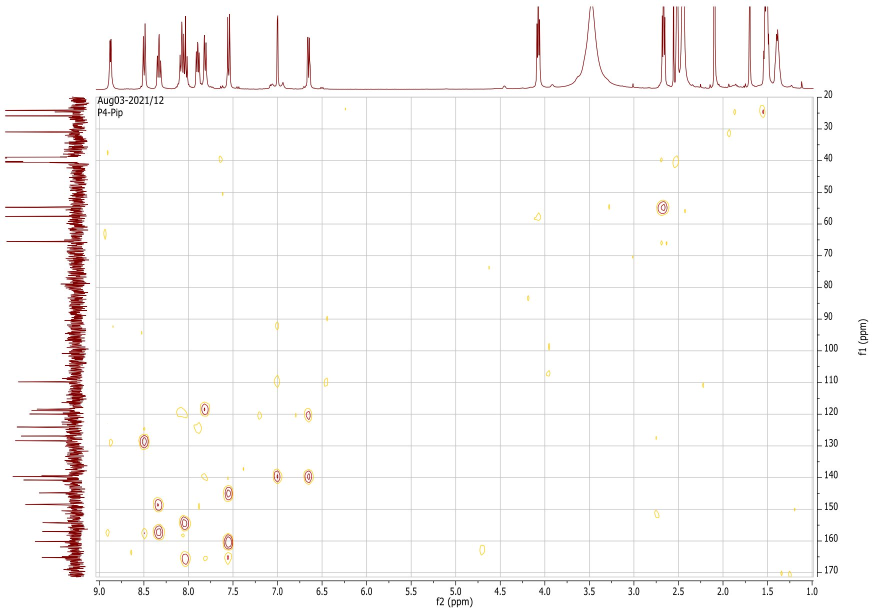


**Figure S7.** HMBC NMR spectra of **3-Pip**.

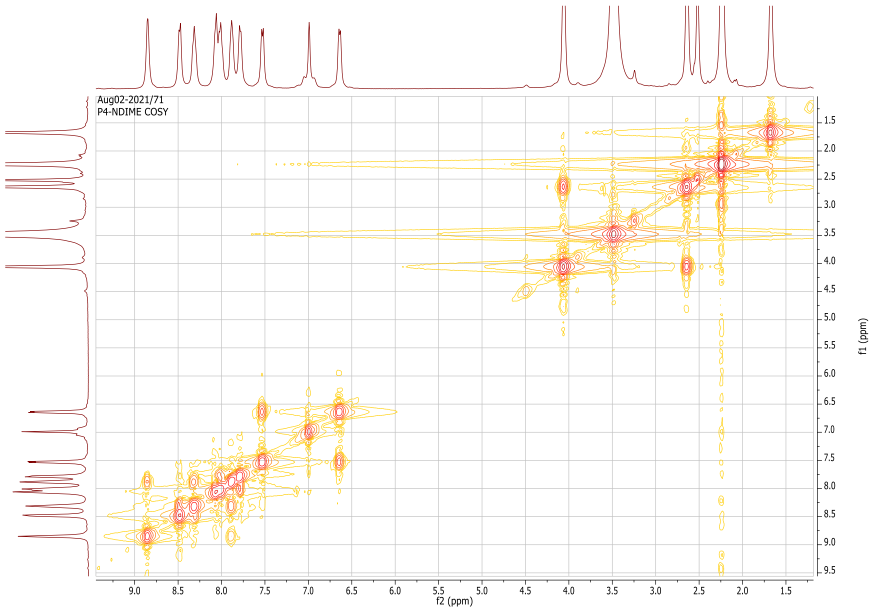


**Figure S8.** COSY NMR spectra of **3-NMe**.

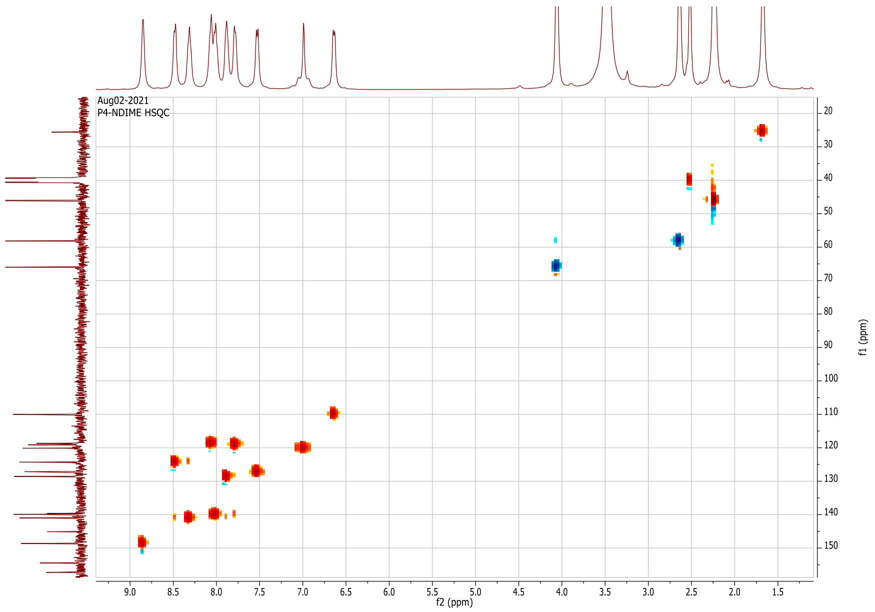


**Figure S9.** HSQC NMR spectra of **3-NMe**.

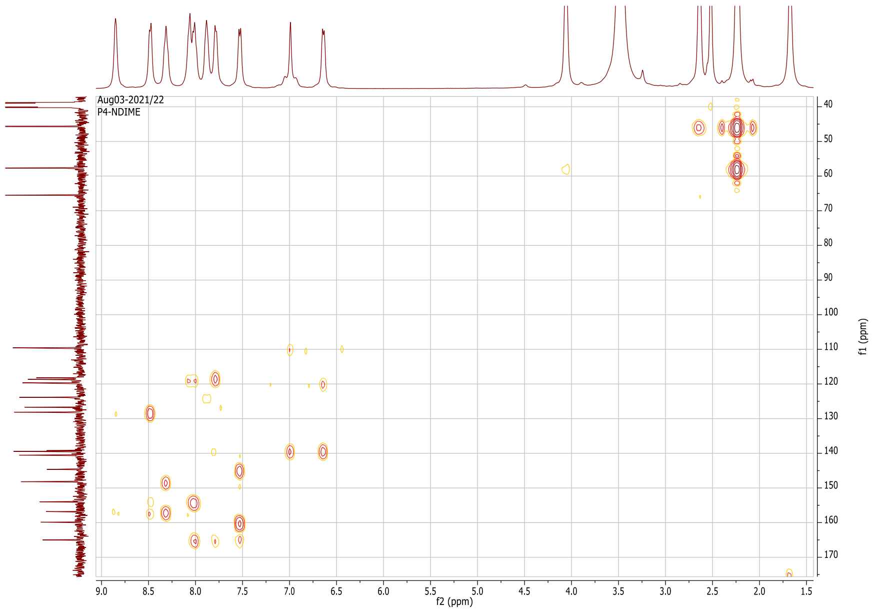


**Figure S10.** HMBC NMR spectra of **3-NMe**.

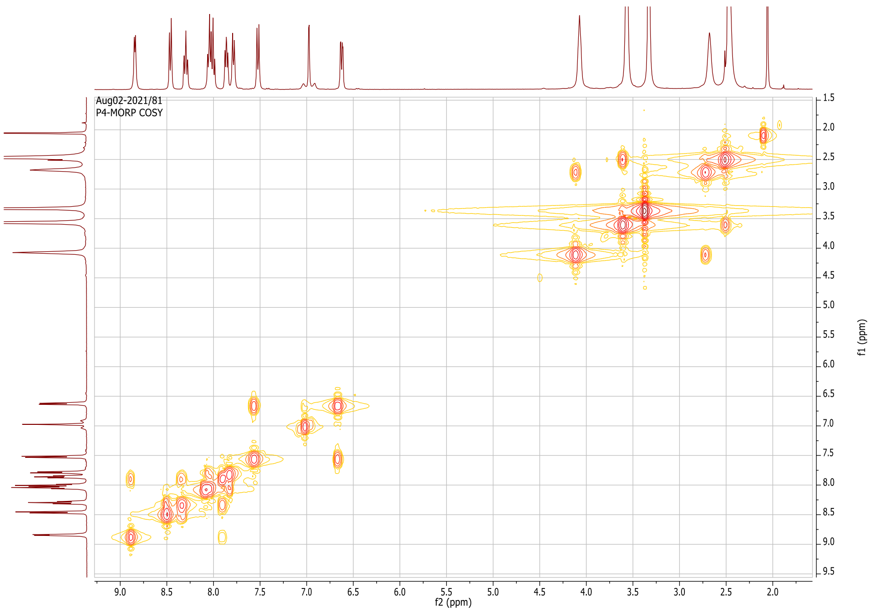


**Figure S11.** COSY NMR spectra of **3-Mor**.

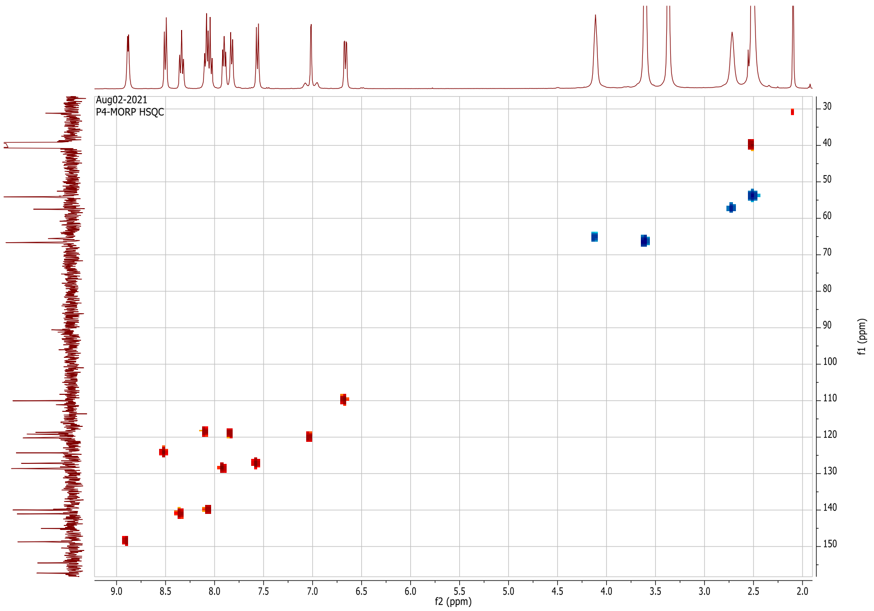


**Figure S12.** HSQC NMR spectra of **3-Mor**.

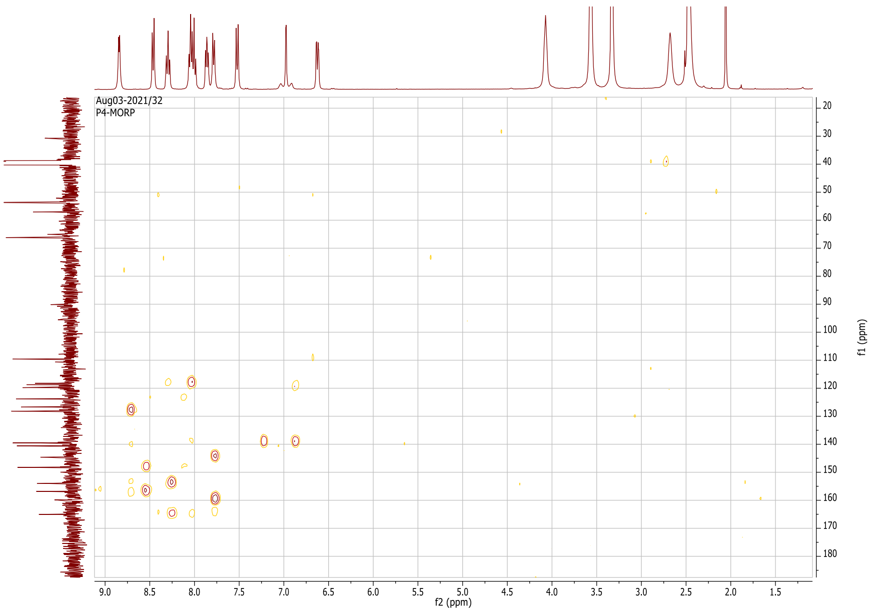


**Figure S13.** HMBC NMR spectra of **3-Mor**.

- NMR spectra of complexes **4-Pip**, **4-NMe** and **4-Mor**

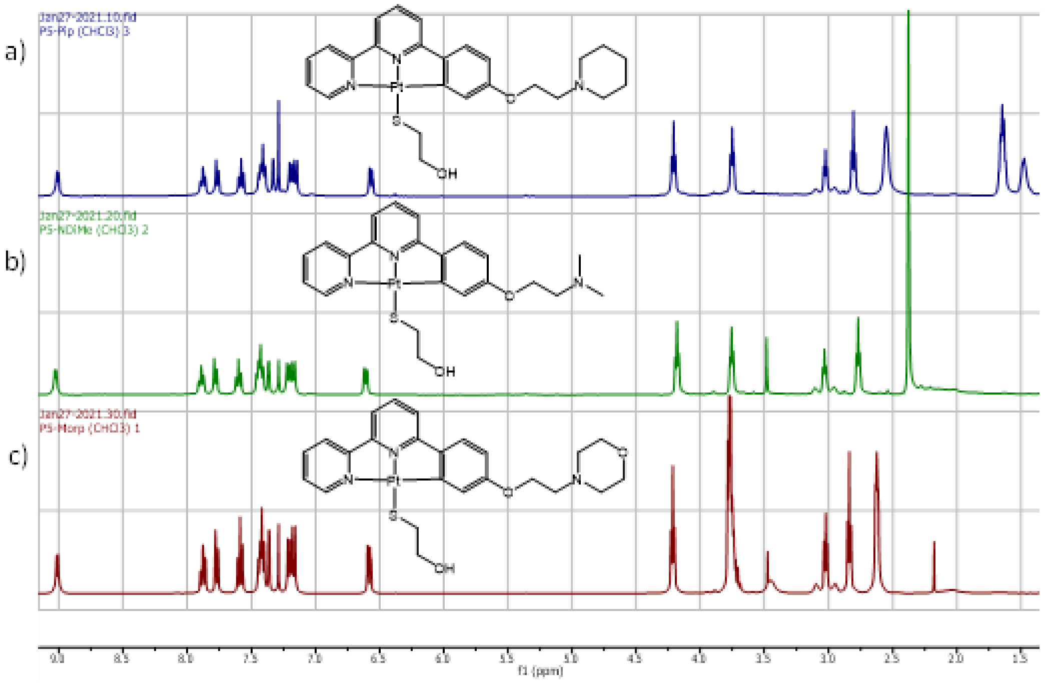


**Figure S14.** $^{1}$H NMR spectra of a) **4-Pip**, b) **4-NMe** and c) **4-Mor**.

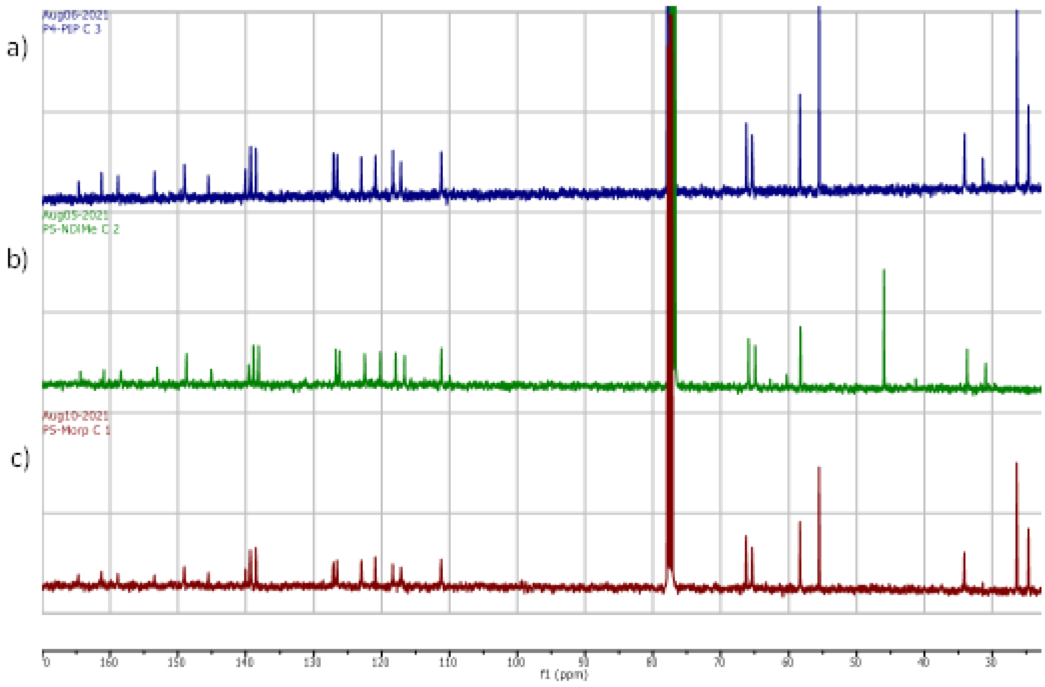


**Figure S15.** $^{13}C$ NMR spectra of a) **4-Pip**, b) **4-NMe** and c) **4-Mor**.

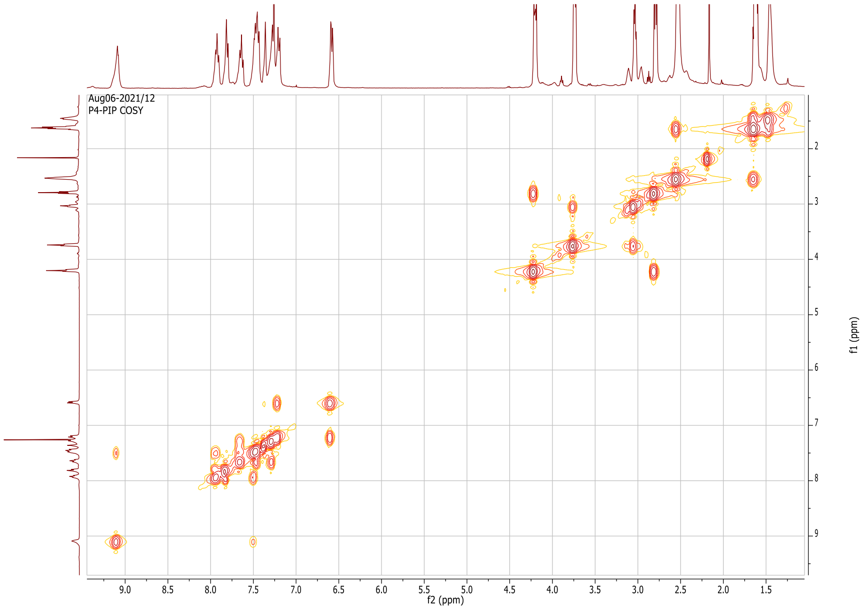


**Figure S16**. COSY NMR spectra of **4-Pip**.

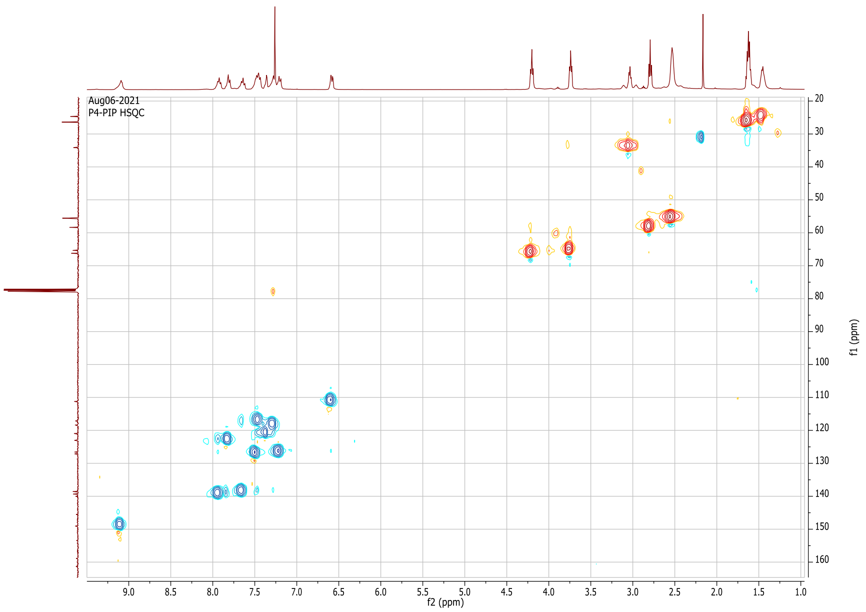


**Figure S17**. HSQC NMR spectra of **4-Pip**.

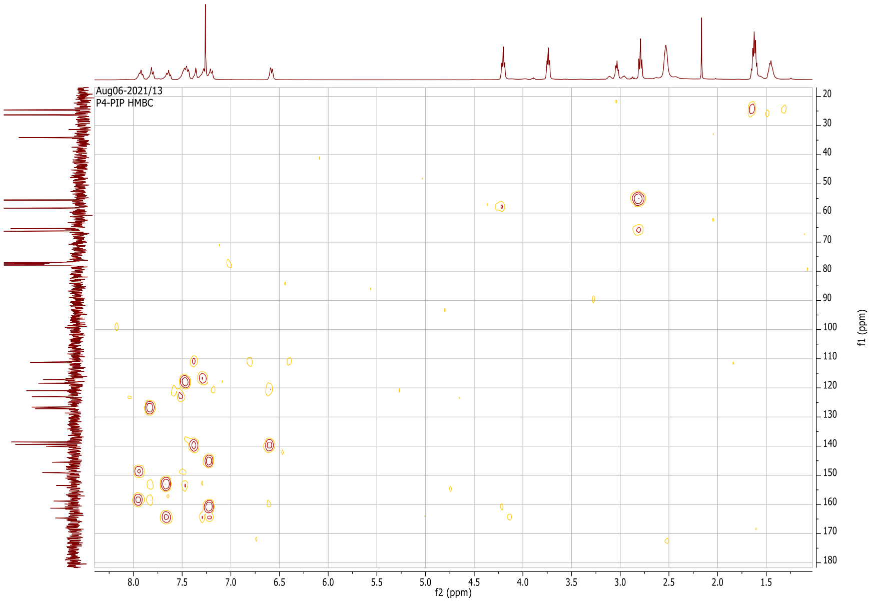


**Figure S18.** HMBC NMR spectra of **4-Pip**.

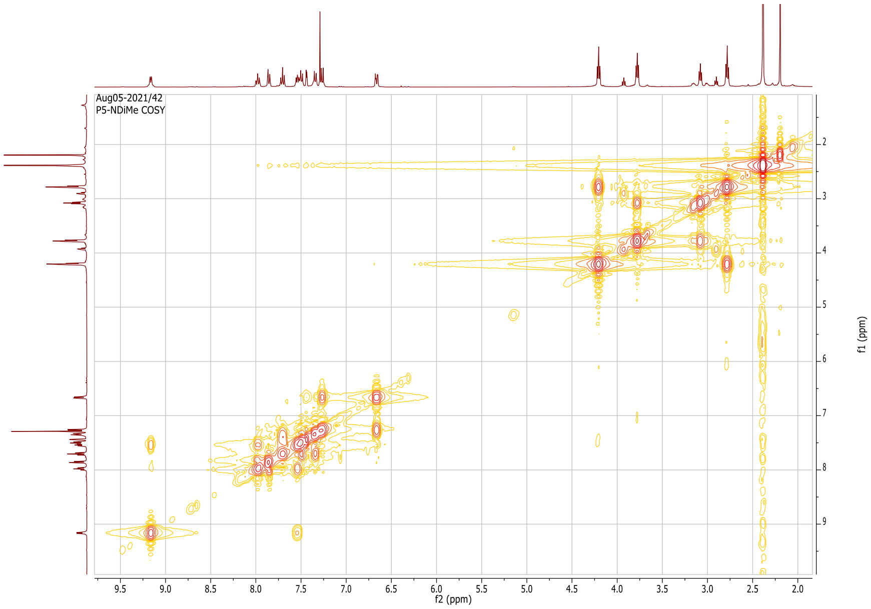


**Figure S19.** COSY NMR spectra of **4-NMe**.

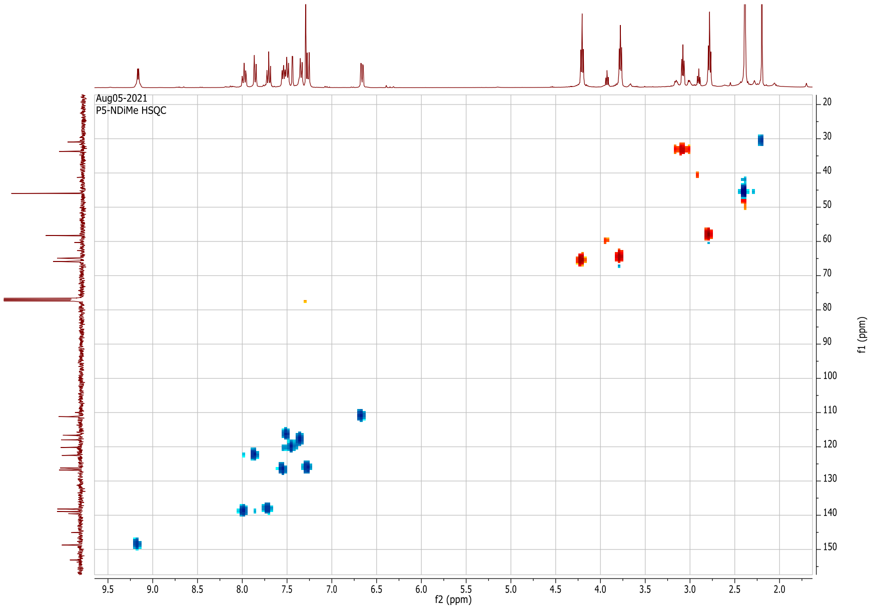


**Figure S20.** HSQC NMR spectra of **4-NMe**.

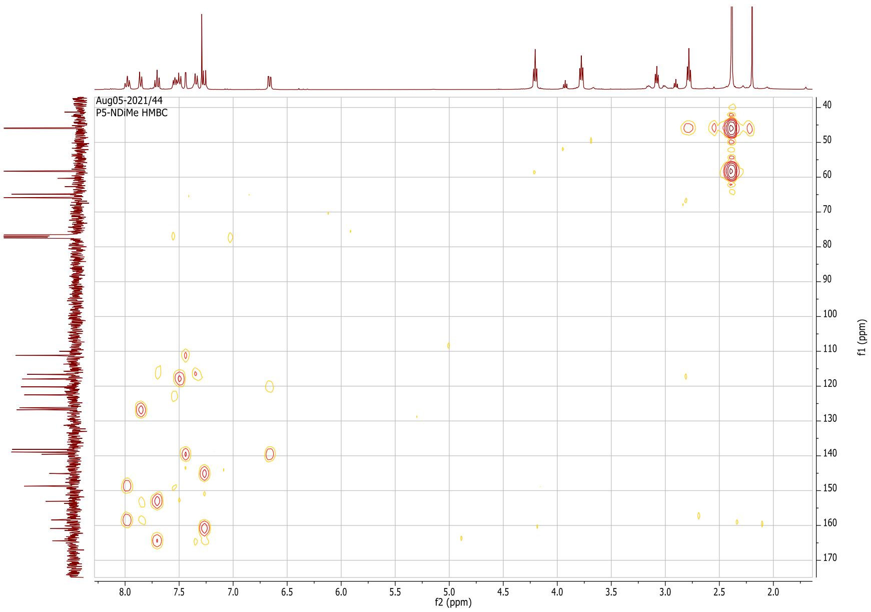


**Figure S21**. HMBC NMR spectra of **4-NMe**.

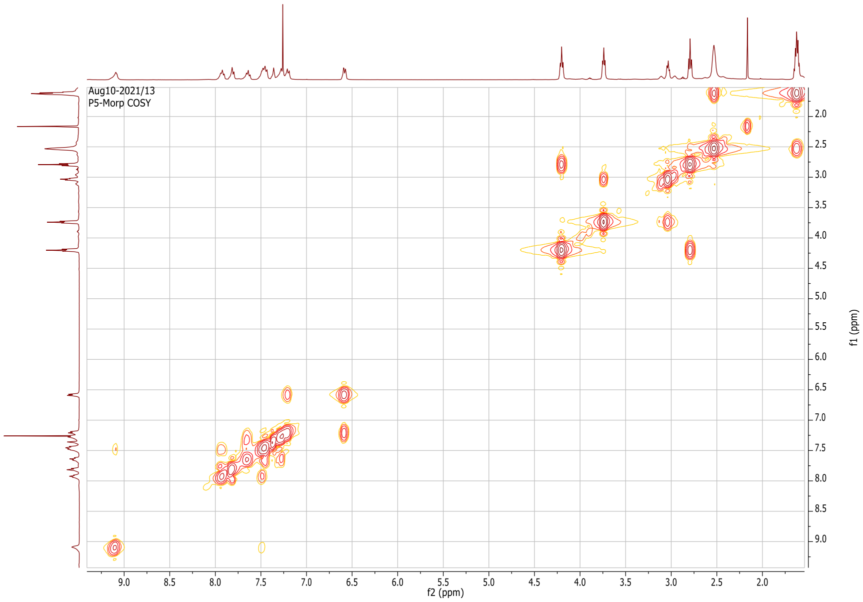


**Figure S22.** COSY NMR spectra of **4-Mor**.

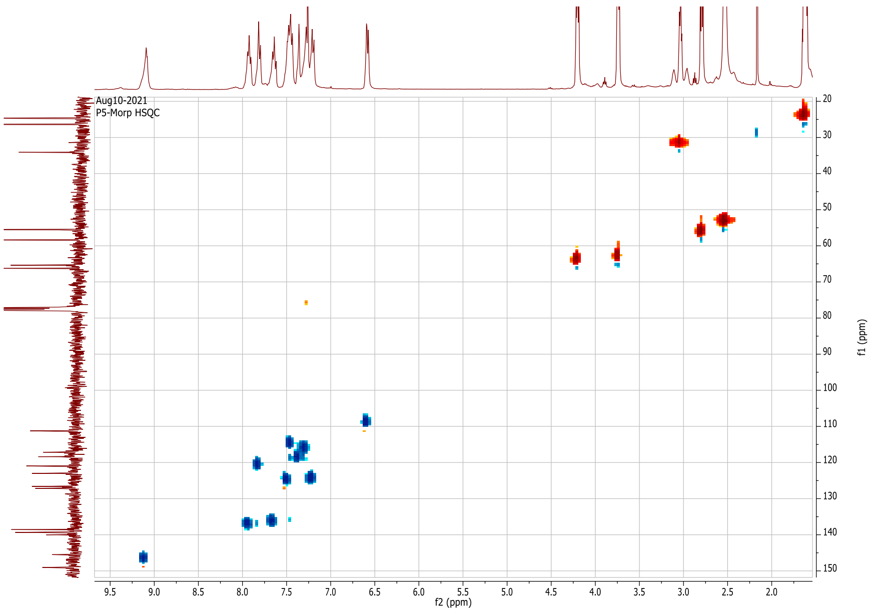


**Figure S23.** HSQC NMR spectra of **4-Mor**.

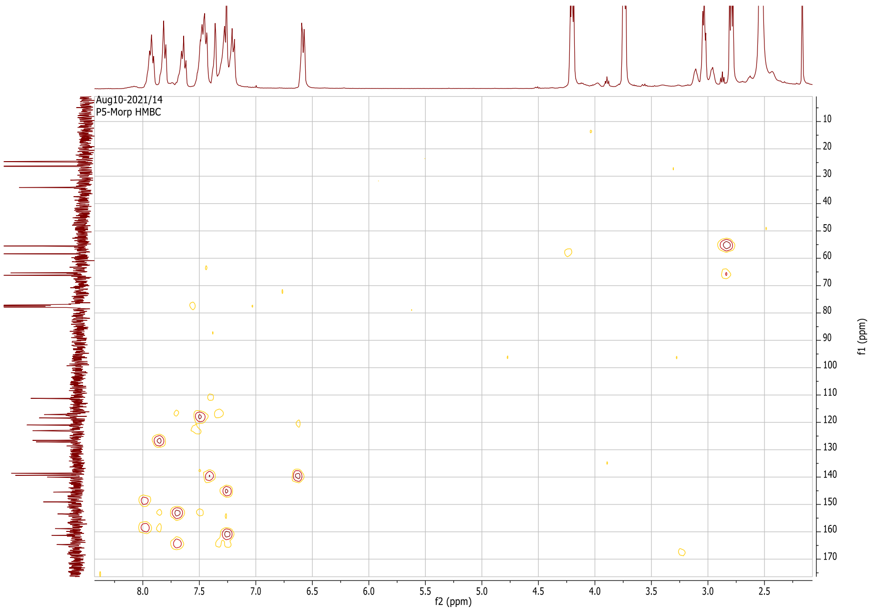


**Figure S24.** HMBC NMR spectra of **4-Mor**.

- Mass spectrometry (ESI).

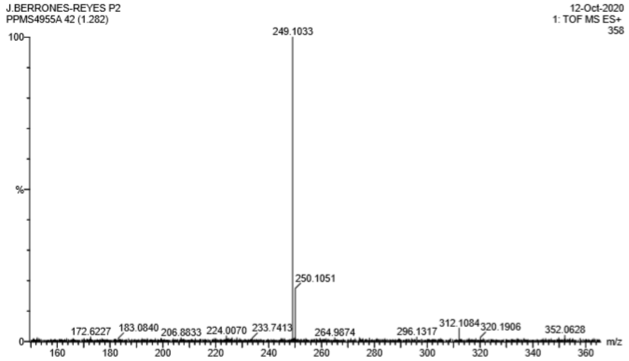


| Mass | Calc. Mass | mDa | PPM | DBE | i-FIT | Formula |
|---|---|---|---|---|---|---|
| Minimum: | | | | -1.5 | | |
| Maximum: | | 5.0 | 5.0 | 1000.0 | | |
| 249.1033 | 249.1028 | 0.5 | 2.0 | 11.5 | 3.9 | C16 H13 N2 O |

**Figure S25.** Mass spectra of **6-(4″-hydroxy-phenyl)-2,2′-bipyridine (1)**.

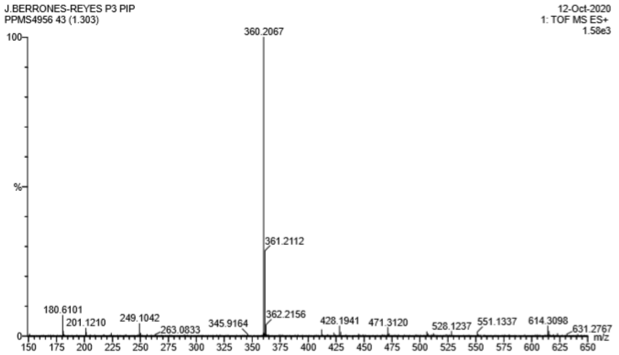


| Mass | Calc. Mass | mDa | PPM | DBE | i-FIT | Formula |
|---|---|---|---|---|---|---|
| Minimum: | | | | -1.5 | | |
| Maximum: | | 5.0 | 5.0 | 1000.0 | | |
| 360.2067 | 360.2076 | -0.9 | -2.5 | 12.5 | 0.4 | C23 H26 N3 O |

**Figure S26.** Mass spectra of ligand **2-Pip**.

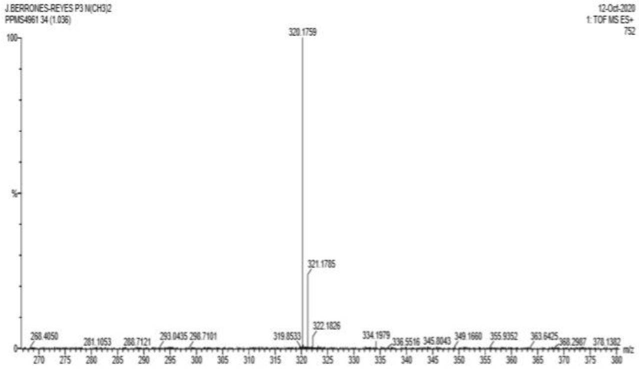


| Mass | Calc. Mass | mDa | PPM | DBE | i-FIT | Formula |
|---|---|---|---|---|---|---|
| Minimum: | | | | -1.5 | | |
| Maximum: | | 5.0 | 10.0 | 1000.0 | | |
| 320.1759 | 320.1763 | -0.4 | -1.2 | 11.5 | 0.6 | C20 H22 N3 O |

**Figure S27.** Mass spectra of ligand **2-NMe**.

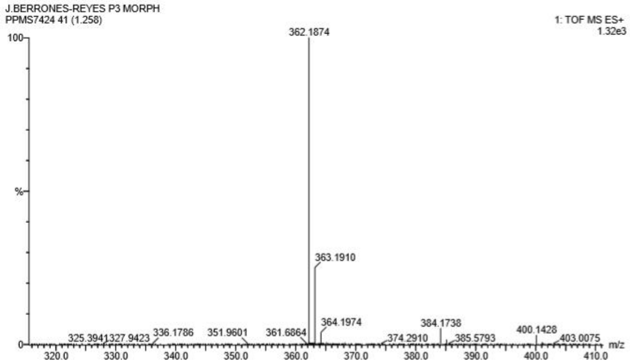


| | | | | | | |
|---|---|---|---|---|---|---|
| Minimum: | | | | -1.5 | | |
| Maximum: | | 5.0 | 7.0 | 50.0 | | |
| Mass | Calc. Mass | mDa | PPM | DBE | i-FIT | Formula |
| 362.1874 | 362.1869 | 0.5 | 1.4 | 12.5 | 0.3 | C22 H24 N3 O2 |

**Figure S28.** Mass spectra of ligand **2-Mor**.

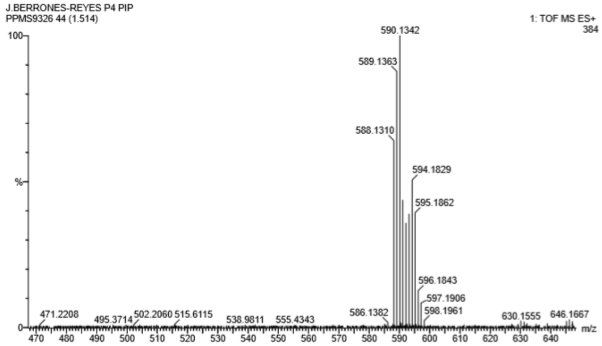


| | | | | | | |
|---|---|---|---|---|---|---|
| Minimum: | | | | -1.5 | | |
| Maximum: | | 5.0 | 10.0 | 50.0 | | |
| Mass | Calc. Mass | mDa | PPM | DBE | i-FIT | Formula |
| 590.1342 | 590.1336 | 0.6 | 1.0 | 13.5 | 1.8 | C23 H25 N3 O Cl 196Pt |
| | 590.1389 | -4.7 | -8.0 | 10.5 | 11.1 | C23 H29 N3 O13 Cl |

**Figure S29.** Mass spectra of complex **3-Pip**.

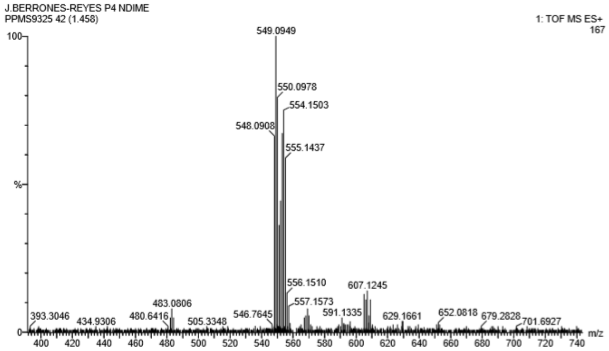


| | | | | | | |
|---|---|---|---|---|---|---|
| Minimum: | | | | -1.5 | | |
| Maximum: | | 5.0 | 10.0 | 50.0 | | |
| Mass | Calc. Mass | mDa | PPM | DBE | i-FIT | Formula |
| 549.0949 | 549.0944 | 0.5 | 0.9 | 13.0 | 12.3 | C20 H20 N3 O Cl 196Pt |
| | 549.0998 | -4.9 | -8.9 | 10.0 | 30.7 | C20 H24 N3 O13 Cl |

**Figure S30.** Mass spectra of complex **3-NMe**.

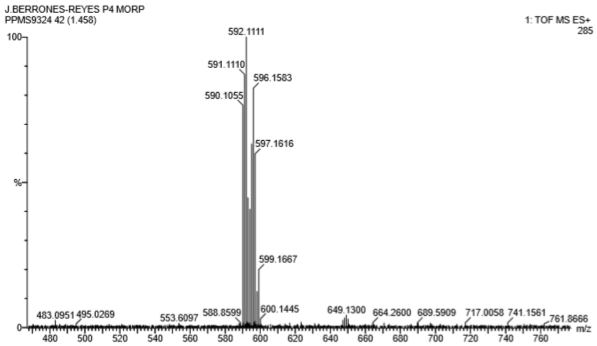


| | | | | | | |
|---|---|---|---|---|---|---|
| Minimum: | | | | -1.5 | | |
| Maximum: | | 5.0 | 10.0 | 50.0 | | |
| Mass | Calc. Mass | mDa | PPM | DBE | i-FIT | Formula |
| 592.1111 | 592.1128 | -1.7 | -2.9 | 13.5 | 0.2 | C22 H23 N3 O2 Cl 196Pt |
| | 592.1069 | 4.2 | 7.1 | 16.0 | 7.6 | C22 H21 N8 O10 Cl |

**Figure S31.** Mass spectra of complex **3-Mor**.

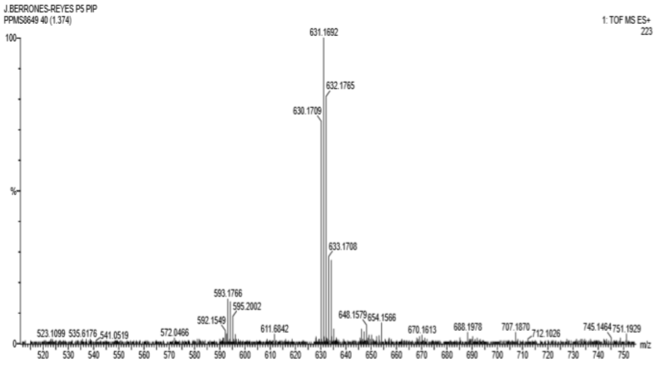


| | | | | | | |
|---|---|---|---|---|---|---|
| Minimum: | | | | -1.5 | | |
| Maximum: | | 5.0 | 7.0 | 50.0 | | |
| Mass | Calc. Mass | mDa | PPM | DBE | i-FIT | Formula |
| 631.1692 | 631.1707 | -1.5 | -2.4 | 13.5 | 0.8 | C25 H30 N3 O2 S 195Pt |

**Figure S32.** Mass spectra of complex **4-Pip**.

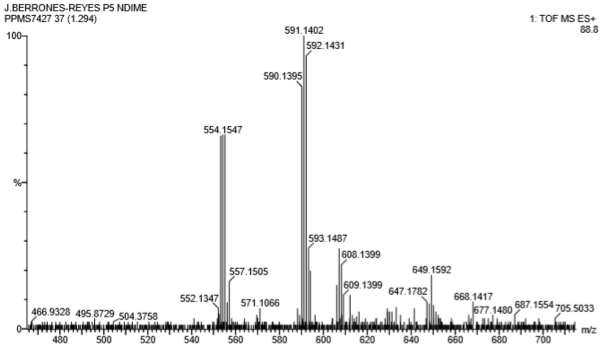


| | | | | | | |
|---|---|---|---|---|---|---|
| Minimum: | | | | -1.5 | | |
| Maximum: | | 5.0 | 7.0 | 50.0 | | |
| Mass | Calc. Mass | mDa | PPM | DBE | i-FIT | Formula |
| 591.1402 | 591.1394 | 0.8 | 1.4 | 12.5 | 0.2 | C22 H26 N3 O2 S 195Pt |
| | 591.1370 | 3.2 | 5.4 | 10.0 | 22.4 | C22 H29 N3 O14 S |

**Figure S33.** Mass spectra of complex **4-NMe**.

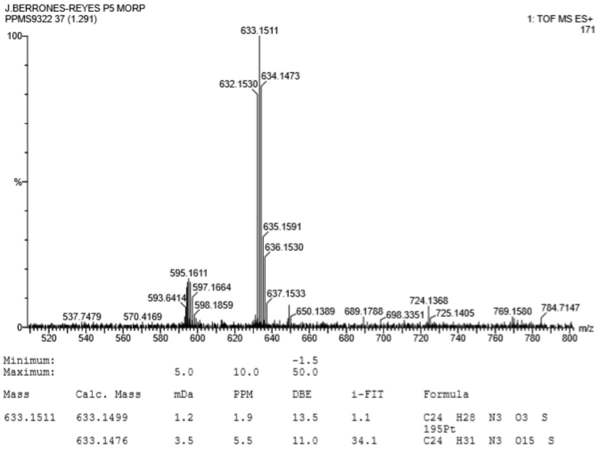


**Figure S34.** Mass spectra of complex **4-Mor**.

**Table S1.** Theoretical and an experimentally observed m/z [M+H$^+$] of the complexes **3-Pip, 3-NMe, 3-Mor, 4-Pip, 4-NMe** and **4-Mor**.

| | Found | Calculated |
|---|---|---|
| **3-Pip** | 590.1342 | 590.1336 |
| **3-NMe** | 549.0949 | 549.0944 |
| **3-Mor** | 592.1111 | 592.1128 |
| **4-Pip** | 631.1692 | 631.1707 |
| **4-NMe** | 591.1402 | 591.1394 |
| **4-Mor** | 633.1511 | 633.1499 |

- Absorption and Emission spectra.

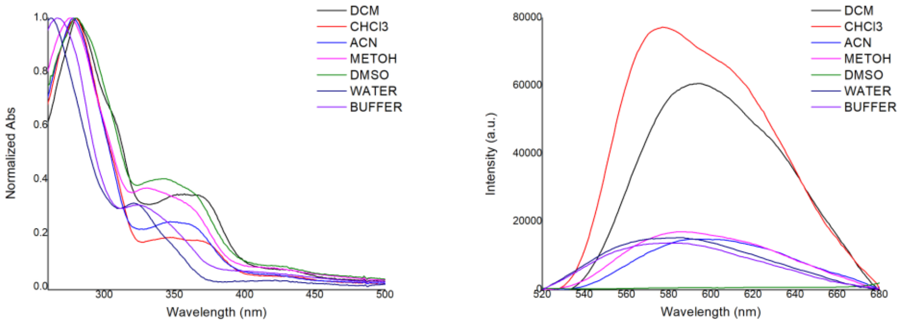


**Figure S35.** Absorption and emission spectra ($\lambda_{ex}$: 350 nm) of **3-Pip**. The shoulder peak in emission seen at ca 600-620 nm in chlorinated solvents is likely to originate from the vibronic coupling of the electronic transition (the energy spacing from the maximum is ca 1000 cm$^{-1}$ ).

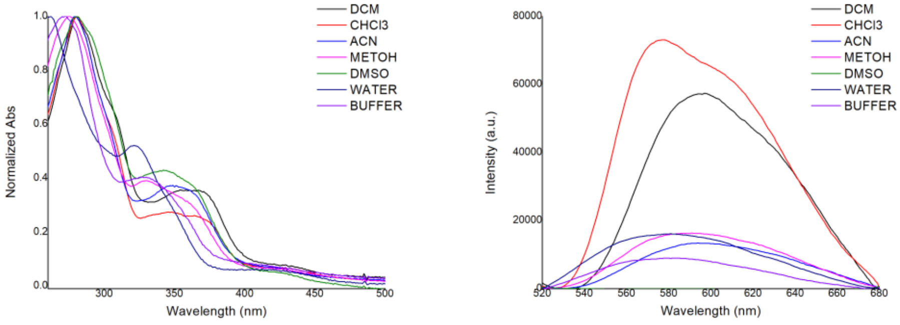


**Figure S36**. Absorption and emission spectra ($\lambda_{ex}$: 350 nm) of **3-NMe**. The shoulder peak in emission seen at ca 600-620 nm in chlorinated solvents is likely to originate from the vibronic coupling of the electronic transition (the energy spacing from the maximum is ca 1000 $cm^{-1}$ ).

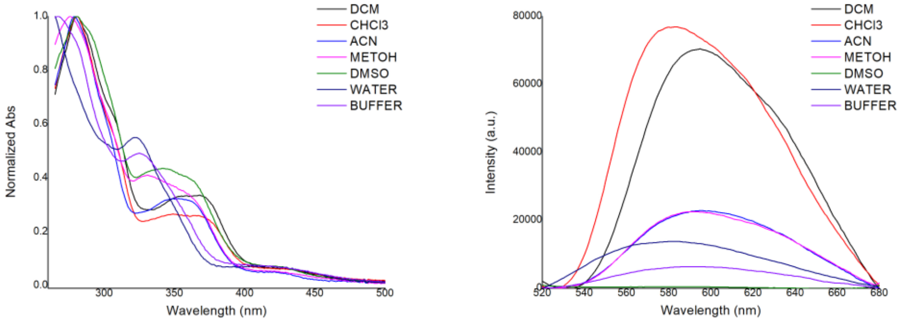


**Figure S37.** Absorption and emission spectra ($\lambda_{ex}$: 350 nm) of **3-Mor**. The shoulder peak in emission seen at ca 600-620 nm in chlorinated solvents is likely to originate from the vibronic coupling of the electronic transition (the energy spacing from the maximum is ca 1000 $cm^{-1}$ ).

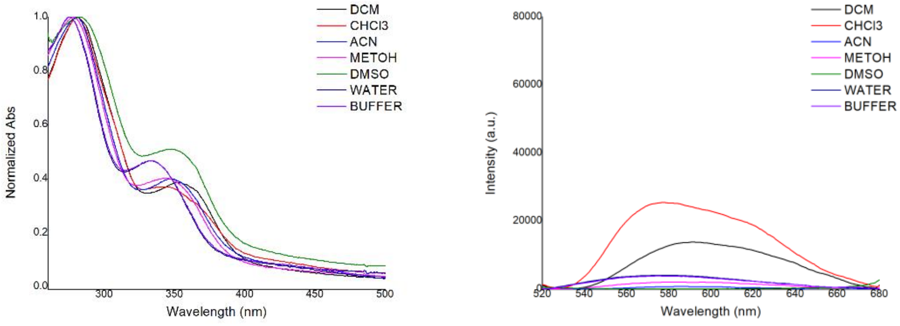


**Figure S38.** Absorption and emission spectra ($\lambda_{ex}$: 350 nm) of **4-Pip**. The shoulder peak in emission seen at ca 600-620 nm in chlorinated solvents is likely to originate from the vibronic coupling of the electronic transition (the energy spacing from the maximum is ca 1000 $cm^{-1}$ ).

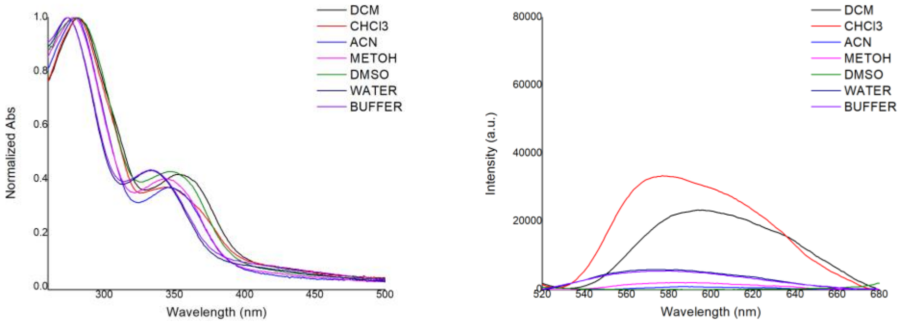


**Figure S39.** Absorption and emission spectra ($\lambda_{ex}$: 350 nm) of **4-NMe**. The shoulder peak in emission seen at ca 600-620 nm in chlorinated solvents is likely to originate from the vibronic coupling of the electronic transition (the energy spacing from the maximum is ca 1000 $cm^{-1}$ ).

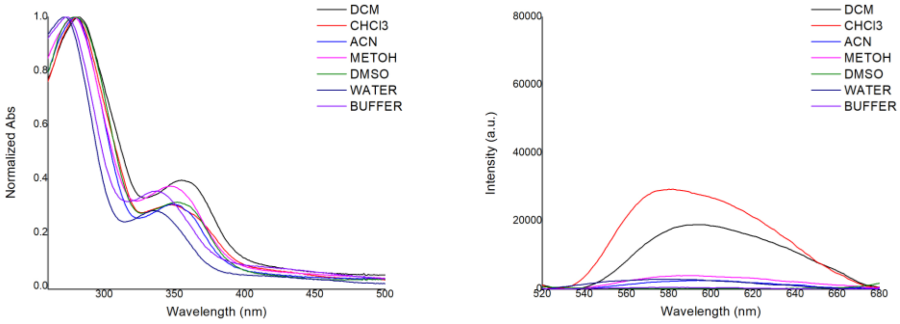


**Figure S40.** Absorption and emission spectra ($\lambda_{ex}$: 350 nm) of **4-Mor**. The shoulder peak in emission seen at ca 600-620 nm in chlorinated solvents is likely to originate from the vibronic coupling of the electronic transition (the energy spacing from the maximum is ca 1000 $cm^{-1}$ ).

**Table S2.** Emission quantum yield values (referenced to quinine) of the complexes **P3-Pip, P3-NMe, P3-Mor, P4-Pip, P4-NMe** and **P4-Mor** in aqueous media, with and without st-DNA. The standard error is 10%.

| | COMPOUND | COMP + DNA |
|---|---|---|
| **3-Pip** | 0.005 | 0.031 |
| **3-NMe** | 0.006 | 0.034 |
| **3-Mor** | 0.002 | 0.025 |
| **4-Pip** | 0.004 | 0.026 |
| **4-NMe** | 0.005 | 0.024 |
| **4-Mor** | 0.001 | 0.023 |

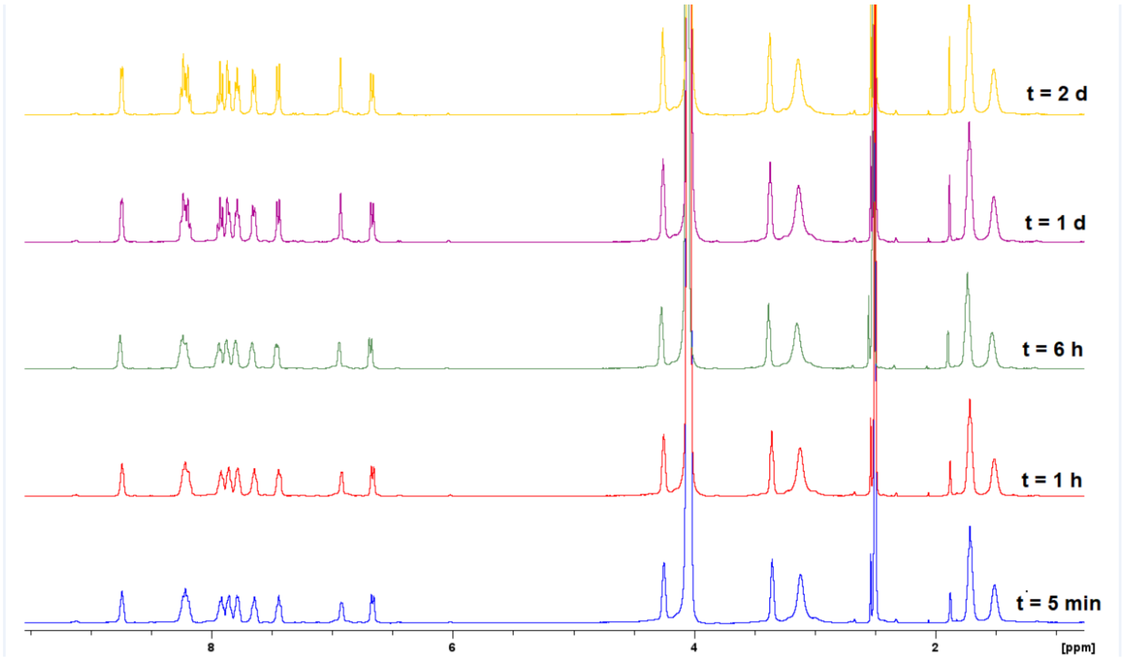


**Figure S41.** $^{1}$H NMR spectra of a freshly prepared solution of **3-pip** in a mixture of DMSO-$d_6$ and $D_2O$ (7:3 ratio) at different times points.

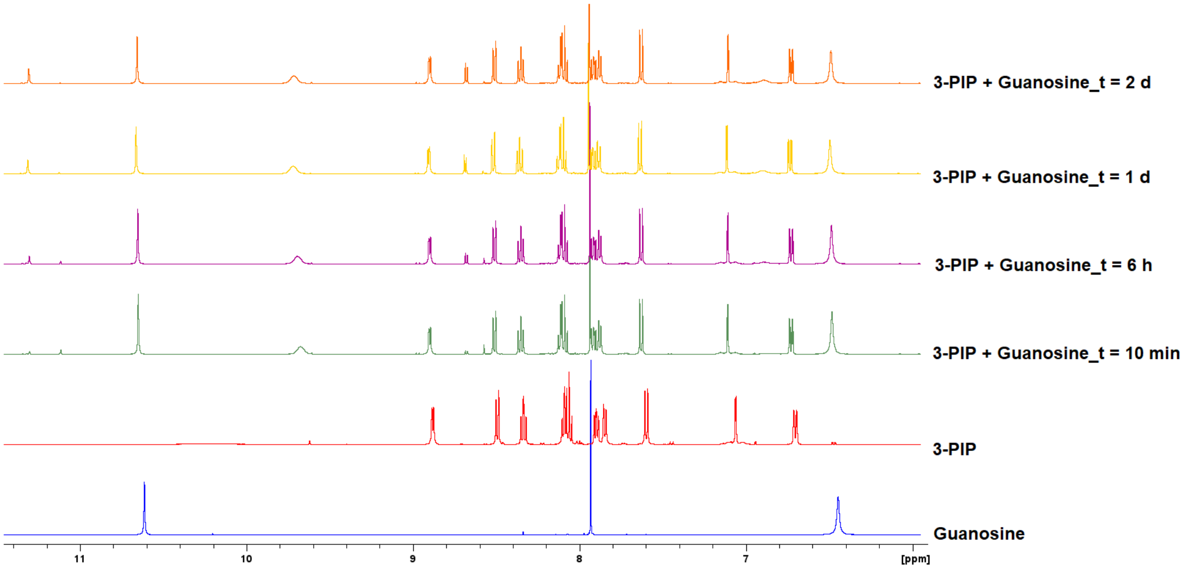


**Figure S42.** $^{1}$H NMR spectra of guanosine, **3-Pip** and **3-Pip**+guanosine over time. Samples dissolved in DMSO-$d_6$ and $D_2O$ (7:3 ratio).

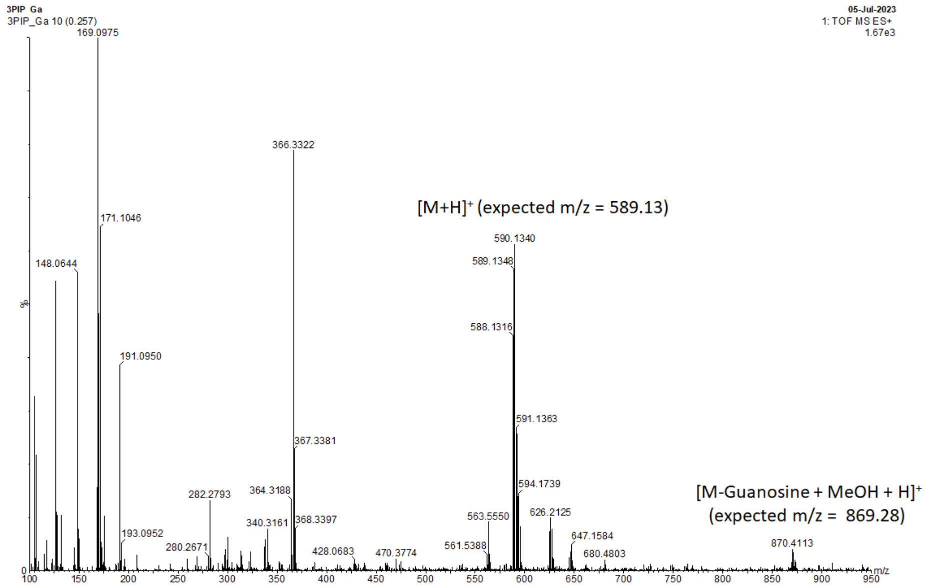


**Figure S43.** Mass spectrum of **3-Pip** + guanosine after they had been incubated for 24 h. The spectrum clearly shows the presence of the unmodified **3-Pip** probe (m/z = 589.13 amu)

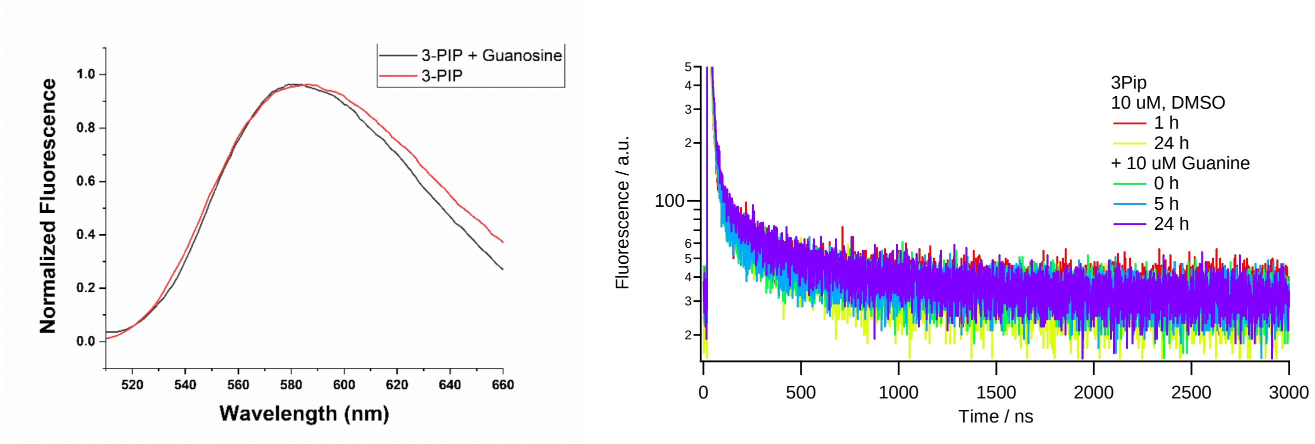


**Figure S44.** (a) Normalised emission spectra of the **3-Pip** + guanosine mixture at time 0 and after 24 h. (b) Time resolved emission decays of the **3-Pip** + guanosine mixture at time 0 and after 5 and 24 h after mixing. The data can be fitted by biexponential decay function with the following parameters:

| | $\tau_1$ / ns ($\alpha_1$, %) | $\tau_2$ / ns ($\alpha_2$, %) | Chi Square |
|---|---|---|---|
| 3-Pip (10 μM) | 7.34 (39.79%) | 236 (60.21%) | 1.25 |
| 3-Pip (10 μM) + Guanine_5 h | 7.32 (69.11%) | 148 (30.89%) | 1.71 |
| 3-Pip (10 μM) + Guanine_24 h | 7.91 (62.12%) | 247 (37.88%) | 1.87 |

This data shown that the emission profile and lifetime of the sample are dominated by the unsubstituted **3-Pip** probe.

(a)

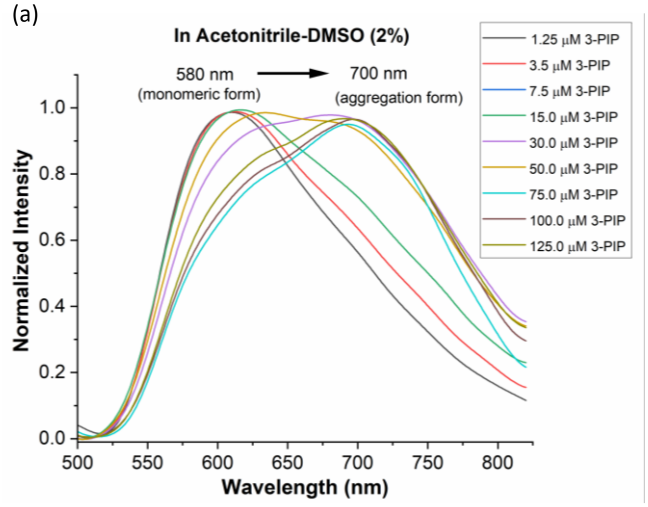


(b)

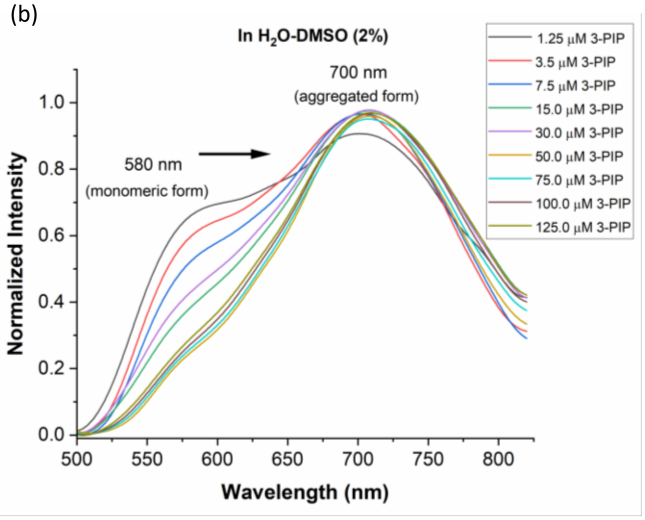


**Figure S45.** Normalised emission spectra of 3-Pip at different concentrations (1.25 to 124 µM) in (a) MeCN/DMSO and (b) H2O/DMSO. It should be noted that at concentrations above 50 µM, a precipitate starts to form (consistent with the aggregation of the probe at higher concentrations).

## DNA binding studies

### *Fluorescence titrations*

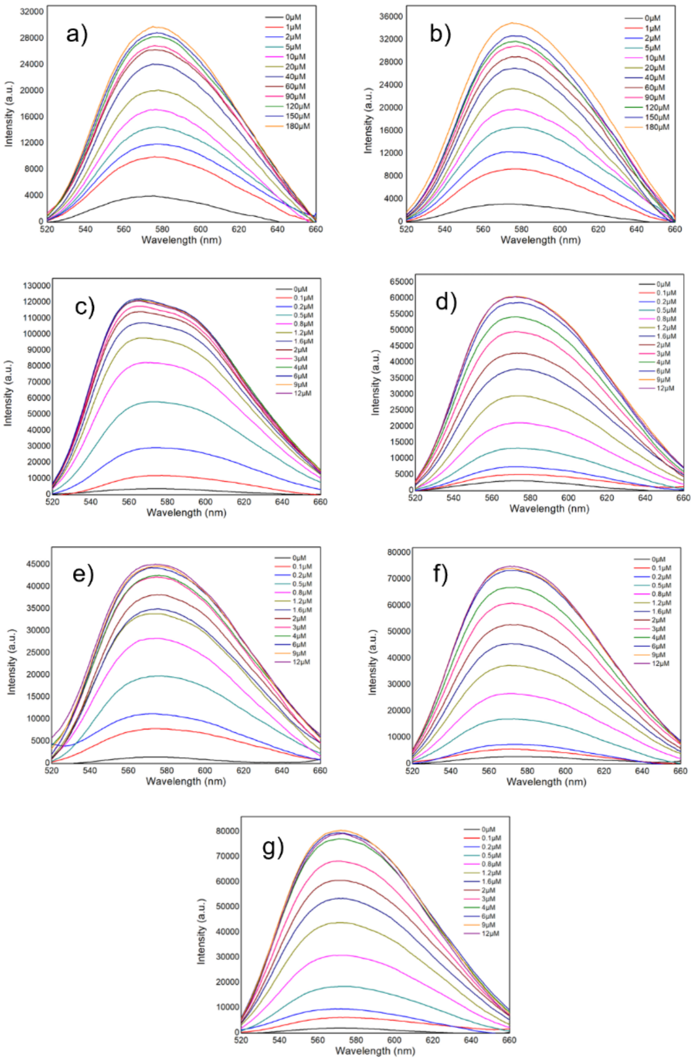


**Figure S46.** Emission spectra of DNA titrations of **3-Pip** with a) ct-DNA, b) st-DNA, c) c-Myc, d) ckit-2, e) HTG4, f) HTelo ($K^+$) and g) HTelo ($Na^+$). $\lambda_{ex}$ = 350 nm. The DNA solutions (both G4 and duplex) used for the experiments described in this figure were prepared in lithium cacodylate buffer at pH 7.3 and supplemented with either $K^+$ of $Na^+$ as described in the experimental details (see above for details).

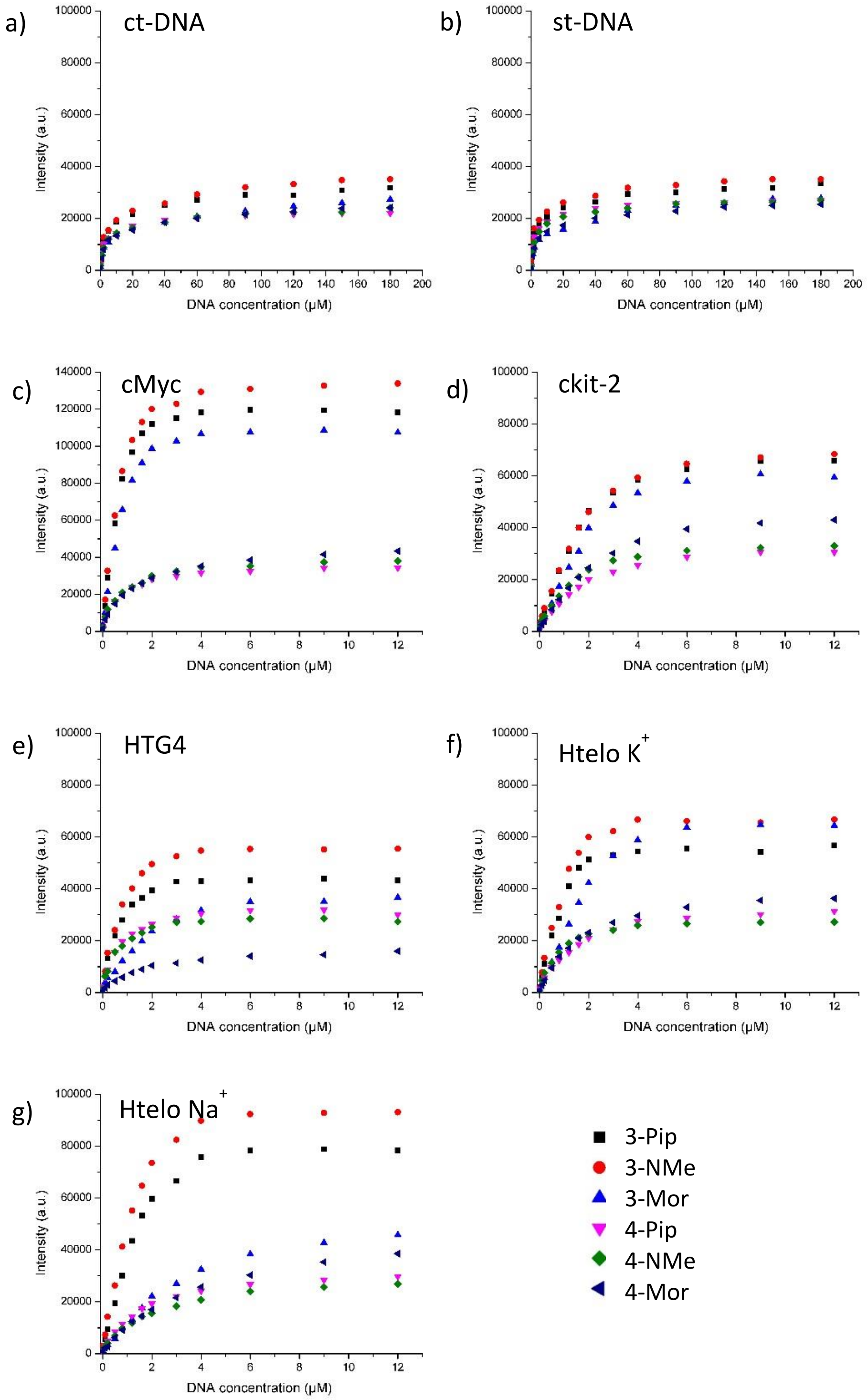


**Figure S47.** DNA binding curves based on emission titrations of **3-Pip**, **3-NMe**, **3-Mor**, **4-Pip**, **4-NMe** and **4-Mor** with a) ct-DNA, b) st-DNA, c) cMyc, d) ckit-2, e) HTG4, f) HTelo ($K^+$) and g) HTelo ($Na^+$). $\lambda_{ex}$ = 350 nm, $\lambda_{em}$ = 580 nm. The DNA solutions (both G4 and duplex) used for the experiments described in this figure were prepared in lithium cacodylate buffer at pH 7.3 and supplemented with either $K^+$ of $Na^+$ as described in the experimental details (see above for details).

**Table S3**. Binding affinity values of Pt complexes ($10^5$ $M^{-1}$)

| | 3-Pip | 3-NMe | 3-Mor | 4-Pip | 4-NMe | 4-Mor |
|---|---|---|---|---|---|---|
| **ct DNA** | 1.25 (±0.08) | 0.9 (±0.23) | 0.74 (±0.08) | 1.79 (±0.24) | 1.19 (±0.10) | 0.94 (±0.21) |
| **st DNA** | 2.03 (±1.39) | 1.76 (±0.09) | 0.71 (±0.39) | 2.13 (±0.46) | 1.48 (±0.12) | 1.01 (±0.18) |
| **cMyc** | 21.65 (±0.64) | 17.61 (±2.87) | 13.68 (±1.56) | 15.04 (±1.18) | 14.43 (±7.18) | 6.09 (±0.91) |
| **ckit 2** | 3.99 (±0.15) | 3.81 (±1.03) | 2.96 (±0.17) | 3.6 (±0.57) | 5.56 (±1.48) | 1.48 (±0.28) |
| **HTG4** | 19.18 (±3.60) | 14.89 (±4.37) | 3.55 (±0.92) | 16.35 (±2.30) | 22.04 (±9.23) | 4.45 (±0.58) |
| **Htelo K** | 11.77 (±1.69) | 10.45 (±1.13) | 2.95 (±0.34) | 4.85 (±0.66) | 12.5 (±0.30) | 3.92 (±0.13) |
| **Htelo Na** | 5.06 (±0.59) | 6.22 (±0.34) | 1.47 (±0.08) | 4.5 (±1.54) | 3.5 (±1.01) | 1.46 (±0.05) |

***CD melting experiments***

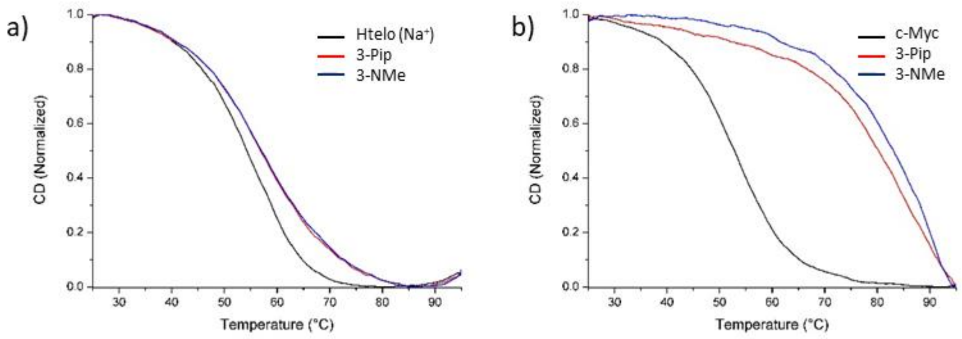


**Figure S48.** CD melting curves of a) HTelo ($Na^+$) G4 DNA (black; 5 µM) and HTelo ($Na^+$) G4 DNA + **3-Pip** (red; 10 µM) and **3-NMe** (blue; 10 µM); b) c-Myc G4 DNA (black; 5 µM) and c-Myc G4 DNA + **3-Pip** (red; 10 µM) and **3-NMe** (blue; 10 µM). The signal at 265 and 295 nm for cMyc and HTelo $Na^+$ respectively was monitored as temperature was increased from 20-95 °C at a rate of °C /min. The DNA solutions used for the experiments described in this figure were prepared in lithium cacodylate buffer at pH 7.3 and supplemented with either $K^+$ of $Na^+$ as described in the experimental details (see above for details).

***In vitro time-correlated single photon counting (TCSPC)***

**Table S4.** Intensity-weighted average lifetime ($\tau_w$) in ns. Lifetimes for the free probes were recorded at a 10 µM while those with DNA, where recorded a 1 µM concentration of probe plus 10 and 100 equivalents of G4 or duplex DNA respectively (in buffer pH 7.4).

| | **Free comp. (aqueous)** | **ct-DNA** | **st-DNA** | **cMyc** | **ckit-2** | **HTG4** | **Htelo K$^+$** | **Htelo Na$^+$** |
|---|---|---|---|---|---|---|---|---|
| **3-Pip** | 75 | 725 | 775 | 1792 | 1901 | 1415 | 1502 | 1423 |
| **3-NMe** | 66 | 758 | 747 | 1514 | 1784 | 1386 | 1373 | 1490 |
| **3-Mor** | 97 | 740 | 784 | 1430 | 1475 | 1235 | 1178 | 1637 |
| **4-Pip** | 267 | 992 | 1092 | 1345 | 1370 | 1331 | 1801 | 1205 |
| **4-NMe** | 269 | 950 | 1144 | 1193 | 1409 | 1242 | 1373 | 1228 |
| **4-Mor** | 147 | 830 | 1042 | 1194 | 1373 | 1149 | 1259 | 1220 |

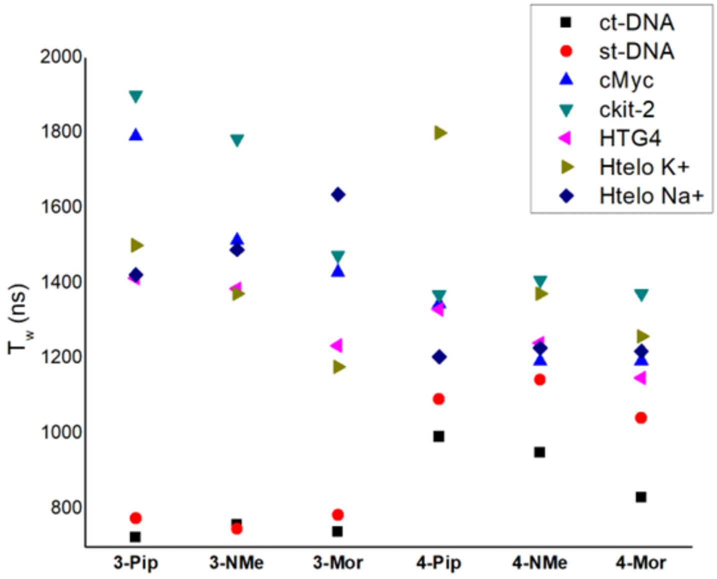


**Figure S49.** Variation of the average lifetime ($\tau_w$) of **3-Pip**, **3-NMe** and **3-Mor, 4-Pip**, **4-NMe** and **4-Morp** in the presence of different G4s and ds-DNA.

## Cell Viability and Fluorescence Microscopy

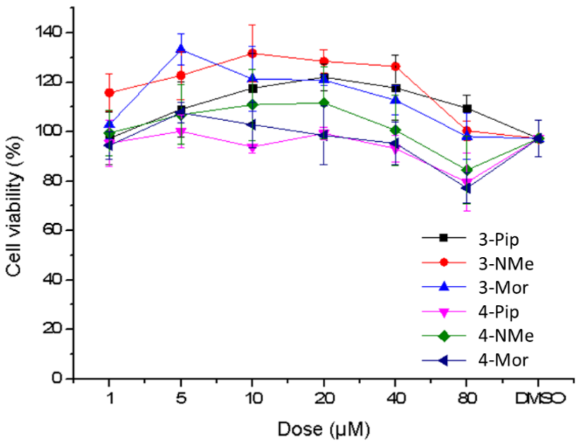


**Figure S50.** MTS cell viability assays after cells being exposed to **3-Pip**, **3-NMe**, **3-Mor**, **4-Pip**, **4-NMe** and **4-Mor** (1-80 µM) for 24 h.

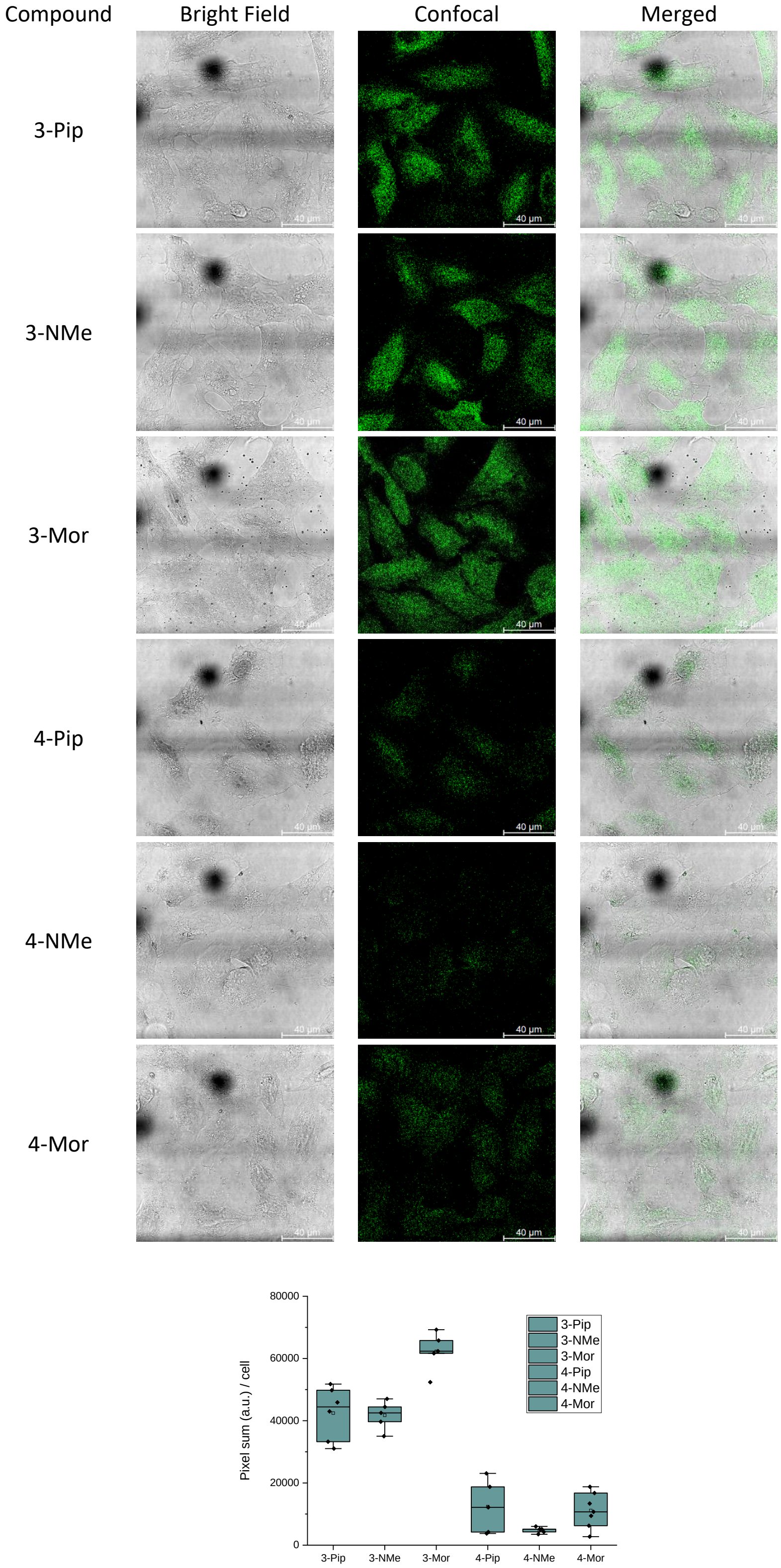


**Figure S51.** Fluorescence imaging experiments of fixed U2OS cells with 80 μM of **3-Pip**, **3-NMe**, **3-Mor, 4-Pip**, **4-NMe** and **4-Mor** (80 μM). $\lambda_{ex}$ = 458nm, $\lambda_{em}$ = 540-620 nm (**3-Mor** aggregates at this concentration).

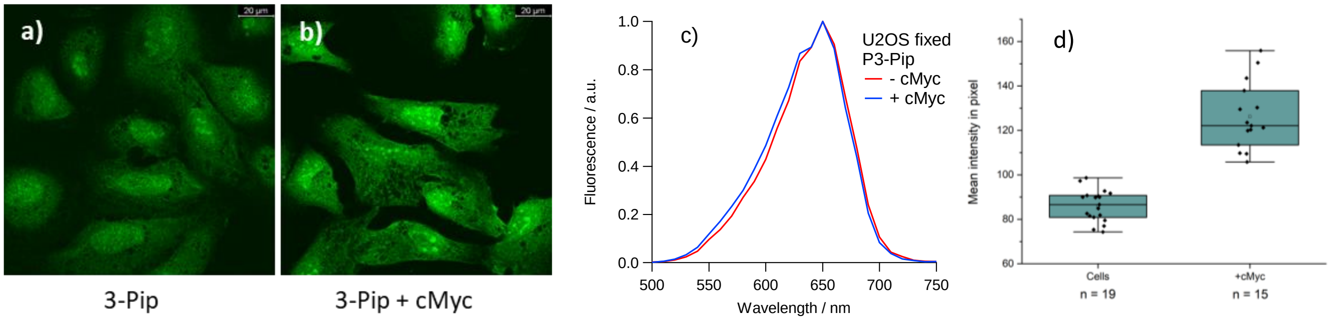


**Figure S52.** Fluorescence imaging experiments of a) fixed U2OS cells with **3-Pip** (80 µM) and b) U2OS cells transfected with c-Myc, fixed and exposed to **3-Pip** (80 µM). c) Emission spectra from cells in a) and b). d) Quantification of the emission intensity from cells treated with 3-Pip only and treated with 3-Pip + transfection of G4 c-Myc (i.e. quantification of images 'a' and 'b').

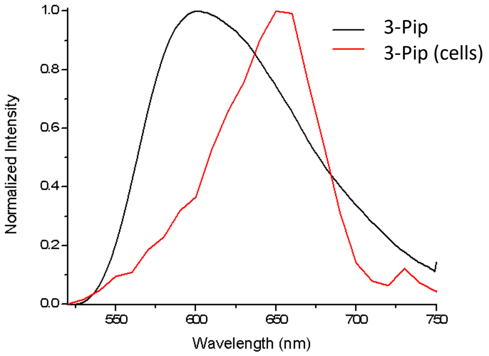


**Figure S53.** Normalized emission spectra of **3-Pip** obtained in the presence of DNA in vitro (black) and **3-Pip** obtained from the cellular images (red).

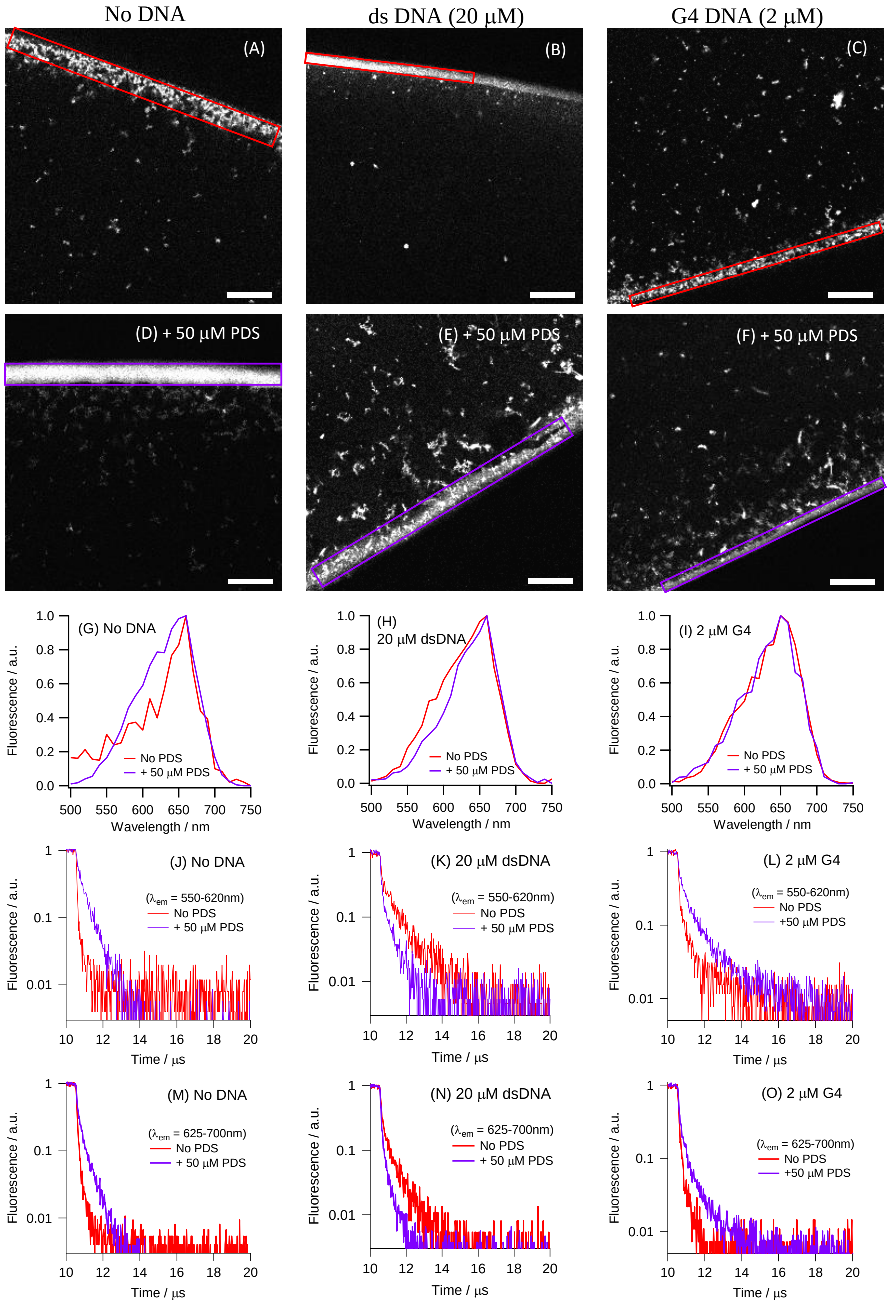


**Fig. S54**. (A-E) Microscopy emission images recorded with 80 μM of **3-Pip** after excitation at 850 nm and detection in 550-620 nm region in the absence (A-C) and presence (D-F) of 50 μM of PDS: (A, D) No DNA; (B, E) + 20 μM of ds DNA; (C, F) + 2 μM of G4 DNA. Red and violet bars highlight regions of interest (ROI) used to extract (G-I) emission spectra and (J-O) time-resolved emission curves in the spectral regions of 550-620 nm (J-L) and 625-700 nm (M-O) in (red) absence and (violet) presence of 50 μM of PDS. Scale bar is 40 μm. At all conditions, the monomer peak (seen as a shoulder at 580 nm) is visible in the spectra, along with an aggregate peak (main peak seen at 650 nm). Therefore, aggregates of **3-Pip** form both in the absence and the presence of G4 and duplex DNA, suggesting that this compound is prone to aggregation at high concentrations. The data

confirms that 650 nm (microscopy) and 700 nm (spectroscopy) emission belong to the **3-Pip** aggregates. The difference in wavelength is due to a different detector sensitivity (and no sensitivity correction is available in microscopy-based spectral measurements).

**Table S5**. Lifetimes and relative amplitudes obtained in the two or three exponential fitting of time resolved phosphorescence curves recorded in the spectral region 550-620 nm and presented in Fig. S49 (J-L).

| | 50 μM PDS | $\tau_1$ / ns ($\alpha_1$, %) | $\tau_2$ / ns ($\alpha_2$, %) | $\tau_3$ / ns ($\alpha_3$, %) |
|---|---|---|---|---|
| No DNA | – | 23 (99.2%) | 244 (0.8%) | – |
| | + | 25 (91.0%) | 290 (6.2%) | 638 (2.8%) |
| + 20 μM dsDNA | – | 108 (96.6%) | 1087 (3.4%) | – |
| | + | 49 (97.4%) | 478 (2.6%) | – |
| + 2 μM G4 | – | 41 (86.1%) | 124 (13.0%) | 624 (0.9%) |
| | + | 48 (92.4%) | 487 (7.1%) | 1827 (0.5%) |

**Table S6**. Lifetimes and relative amplitudes obtained in the two or three exponential fitting of kinetic curves recorded in the spectral region 625-700 nm and presented in Fig. S50 (M-O).

| | 50 μM PDS | $\tau_1$ / ns ($\alpha_1$, %) | $\tau_2$ / ns ($\alpha_2$, %) | $\tau_3$ / ns ($\alpha_3$, %) |
|---|---|---|---|---|
| No DNA | – | 30 (98.3%) | 250 (1.7%) | – |
| | + | 24 (92.7%) | 194 (5.7%) | 580 (1.6%) |
| + 20 μM dsDNA | – | 65 (98.0%) | 728 (2.0%) | – |
| | + | 40 (97.7%) | 326 (2.3%) | – |
| + 2 μM G4 | – | 43 (74.7%) | 94 (24.4%) | 461 (0.9%) |
| | + | 53 (82.4%) | 153 (16.1%) | 808 (1.5%) |

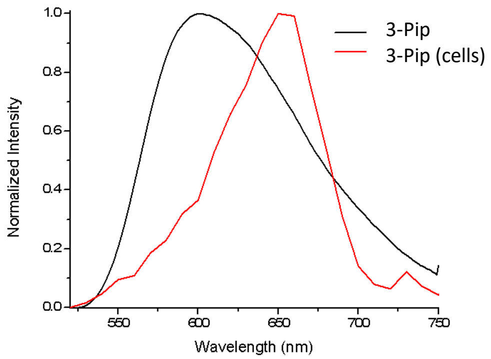


**Figure S55.** Normalized emission spectra obtained from the cellular images of U2OS cells incubated with 80 µM (black) and 20 µM (red) **3-Pip**. A clear shoulder at 580 nm (monomer) is seen in the red spectrum, giving evidence for the partial disaggregation of **3-Pip** at lower incubation concentration.

**Table S7** - Lifetimes and relative amplitudes obtained in the three-exponential fitting of kinetic curves presented in Fig. 4 (main text).

| | $\tau_1$ / ns ($\alpha_1$, %) | $\tau_2$ / ns ($\alpha_2$, %) | $\tau_3$ / ns ($\alpha_3$, %) |
|---|---|---|---|
| Cytosol | 59 (87.2%) | 385 (11.3%) | 1112 (1.5%) |
| Nuclei | 65 (89.7%) | 513 (9.1%) | 1521 (1.2%) |
| Nucleoli | 72 (87.2%) | 671 (9.0%) | 1927 (0.7%) |

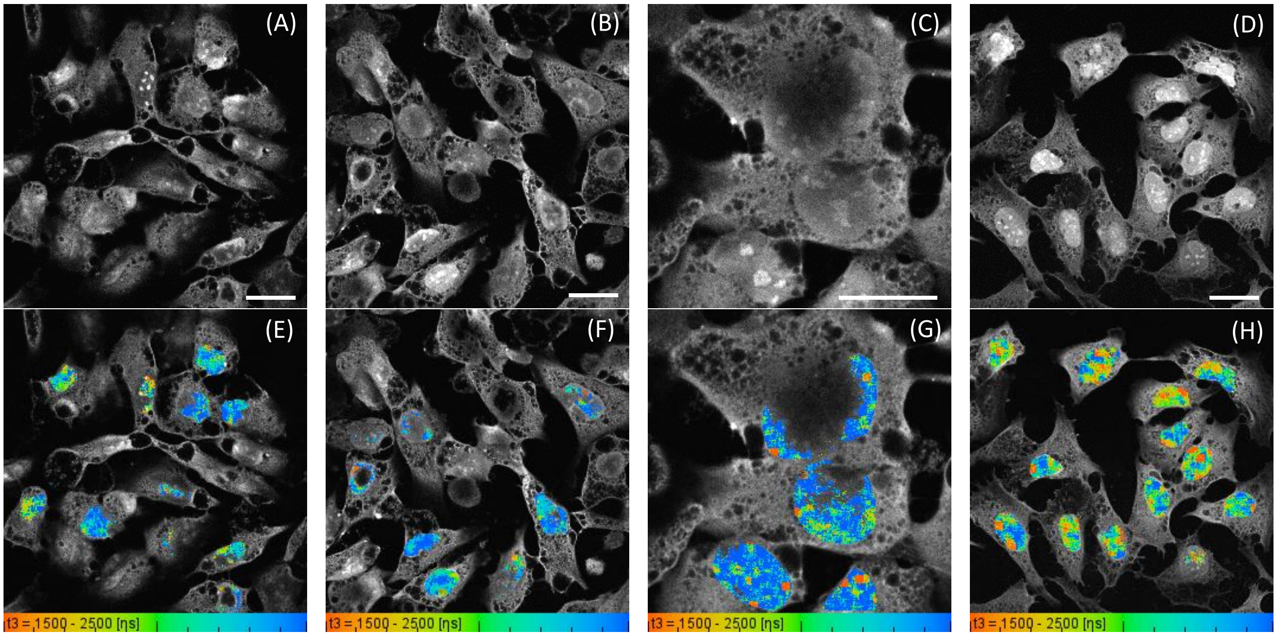


**Figure S56**. (A-D) Emission intensity and (E-H) PLIM images recorded for PFA-fixed U2OS cells stained with 80 μM of **3-Pip** and 50 μM of PDS after excitation at 850 nm and detection in 550-620 nm region. (E-H): The segmentation of nuclei. The scale bar is 40 μm. A clear increase in the observed lifetime is observed upon addition of PDS, see Table S8 for the triexponential fitting of the data.

**Table S8**. Lifetimes and relative amplitudes obtained in the three-exponential fitting of kinetic traces averaged over nuclear selection in Figures 5 (main text) and S52 (ESI). A clear increase in the amplitude $\alpha_3$ of the longest lifetime component ($\tau_3$) is observed upon addition of PDS, compare with the Table S7 data.

| | $\tau_1$ / ns ($\alpha_1$, %) | $\tau_2$ / ns ($\alpha_2$, %) | $\tau_3$ / ns ($\alpha_3$, %) |
|---|---|---|---|
| #1 | 133 (83.4%) | 910 (13.2%) | 2200 (3.4%) |
| #2 | 149 (79.7%) | 876 (16.3%) | 2038 (4.0%) |
| #3 | 98 (80.3%) | 741 (15.8%) | 2062 (4.0%) |
| #4 | 101 (86.9%) | 763 (9.7%) | 1872 (1.0%) |
| #5 | 128 (84.6%) | 986 (12.7%) | 2353 (2.6%) |

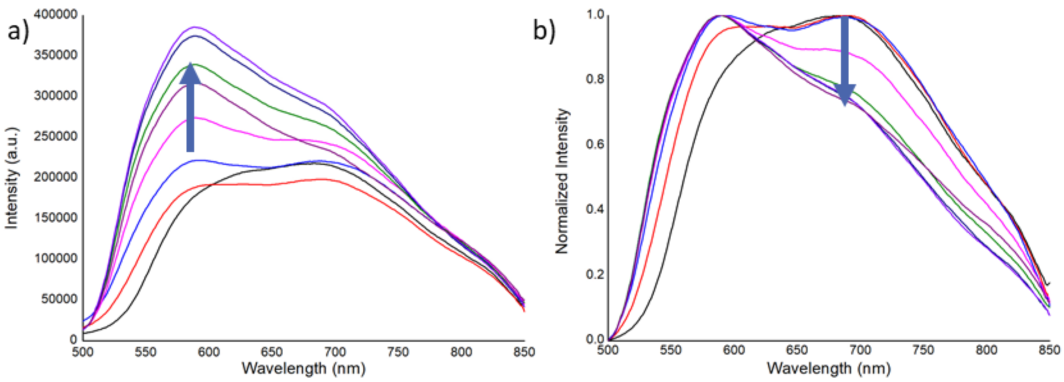


**Figure S57.** a) Not normalized and b) normalized emission spectra of **3-Pip** (50 µM) upon interaction with *HTG4* (2 µM) in the presence of PDS (50 µM), recorded at different time intervals between 0 and 3.5 hours. The spectral change observed (a relative and absolute increase of the 580 nm band intensity, shown by the arrow) is consistent with partial disaggregation of **3-Pip** in the presence of PDS with time.

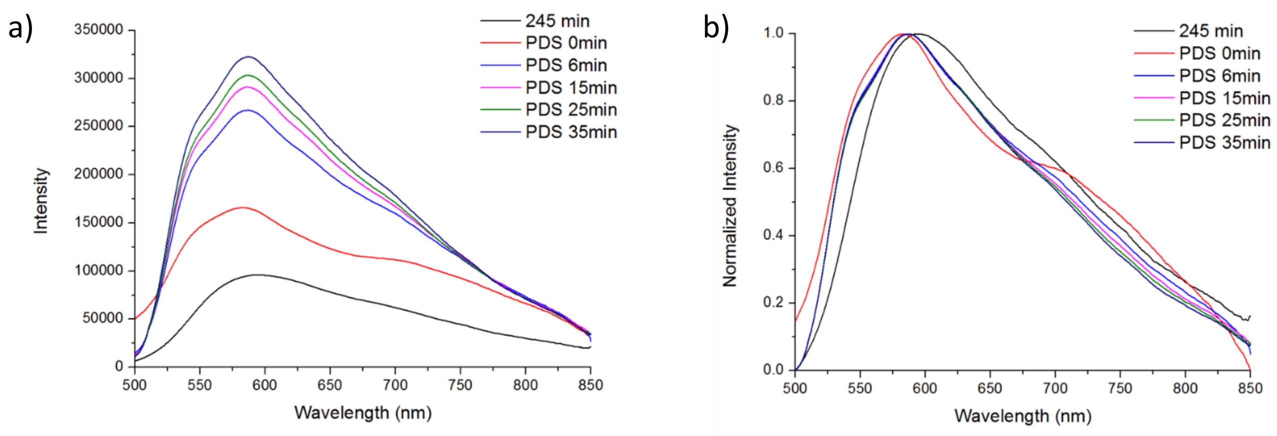


**Figure S58.** a) Not normalized and b) normalized emission spectra of **3-Pip** (50 µM) upon interaction with stDNA (20 µM) in the presence of PDS (50 µM), recorded at different time points (note, PDS was added after DNA and 3-Pip had been incubated for 4 h).

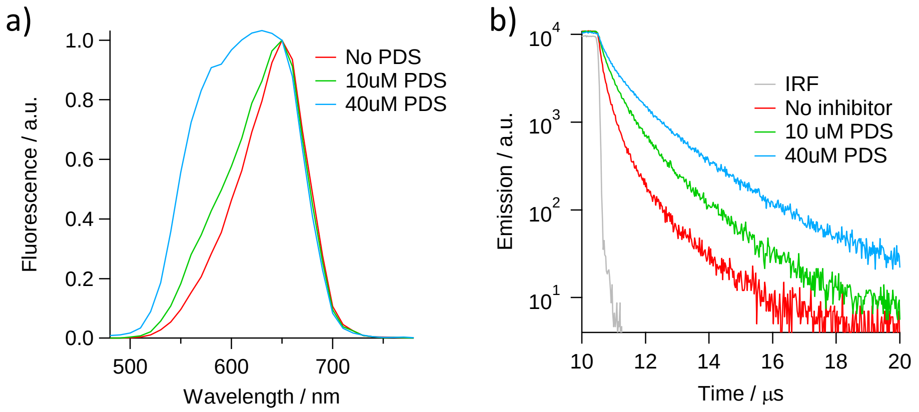


**Figure S59.** a) Normalized emission spectra of **3-Pip** (80 µM) and in the presence of a strong G4 DNA binder PDS at concentrations of 10 µM (green) and 40 µM (blue) obtained from cellular images of fixed U2OS cells, excited at 850 nm; (b) Normalised averaged time resolved phosphorescence decays corresponding to segmented nuclei for various PDS concentrations.
